# Evaluation of the significance of cell wall polymers in flax infected with a pathogenic strain of *Fusarium oxysporum*

**DOI:** 10.1186/s12870-016-0762-z

**Published:** 2016-03-22

**Authors:** Wioleta Wojtasik, Anna Kulma, Lucyna Dymińska, Jerzy Hanuza, Magdalena Czemplik, Jan Szopa

**Affiliations:** Faculty of Biotechnology, University of Wroclaw, Przybyszewskiego 63/77, 51-148 Wroclaw, Poland; Department of Bioorganic Chemistry, Institute of Chemistry and Food Technology, Faculty of Economics and Engineering, University of Economics, Komandorska 118/120, 50-345 Wroclaw, Poland; Institute of Low Temperatures and Structure Research, Polish Academy of Sciences, Okolna 2, 50-422 Wroclaw, Poland; Faculty of Natural Sciences, University of Wroclaw, Przybyszewskiego 63/77, 51-148 Wroclaw, Poland; Department of Genetics, Plant Breeding and Seed Production, Faculty of Life Sciences and Technology, Wroclaw University of Environmental and Plant Sciences, Plac Grunwaldzki 24A, 53-363 Wroclaw, Poland

**Keywords:** Flax, *Fusarium oxysporum*, Infection, Cell wall polymers

## Abstract

**Background:**

*Fusarium oxysporum* infection leads to *Fusarium*-derived wilt, which is responsible for the greatest losses in flax (*Linum usitatissimum*) crop yield. Plants infected by *Fusarium oxysporum* show severe symptoms of dehydration due to the growth of the fungus in vascular tissues. As the disease develops, vascular browning and leaf yellowing can be observed. In the case of more virulent strains, plants die. The pathogen’s attack starts with secretion of enzymes degrading the host cell wall. The main aim of the study was to evaluate the role of the cell wall polymers in the flax plant response to the infection in order to better understand the process of resistance and develop new ways to protect plants against infection. For this purpose, the expression of genes involved in cell wall polymer metabolism and corresponding polymer levels were investigated in flax seedlings after incubation with *Fusarium oxysporum*.

**Results:**

This analysis was facilitated by selecting two groups of genes responding differently to the infection. The first group comprised genes strongly affected by the infection and activated later (phenylalanine ammonia lyase and glucosyltransferase). The second group comprised genes which are slightly affected (up to five times) and their expression vary as the infection progresses. *Fusarium oxysporum* infection did not affect the contents of cell wall polymers, but changed their structure.

**Conclusion:**

The results suggest that the role of the cell wall polymers in the plant response to *Fusarium oxysporum* infection is manifested through changes in expression of their genes and rearrangement of the cell wall polymers. Our studies provided new information about the role of cellulose and hemicelluloses in the infection process, the change of their structure and the expression of genes participating in their metabolism during the pathogen infection. We also confirmed the role of pectin and lignin in this process, indicating the major changes at the mRNA level of lignin metabolism genes and the loosening of the pectin structure.

**Electronic supplementary material:**

The online version of this article (doi:10.1186/s12870-016-0762-z) contains supplementary material, which is available to authorized users.

## Background

Flax (*Linum usitatissimum*) is a unique plant which is a valuable source of fibre and oil. Flax raw materials are applicable in many industrial branches: medicine, pharmacy and cosmetics. It is estimated that around 20 % of flax cultivation loss is a result of fusariosis. These diseases caused by *Fusarium* species fungi contribute to the lowering of yield, grain and fibre quality. The highest pathogenicity towards flax was exhibited by *F. oxysporum* f. sp. *linii*, which causes flax wilt [1, 2].

The plant cell wall is the first physical barrier to pathogen infection. During the first stages of infection, pathogens secrete enzymes that degrade the cell wall: pectinases, cellulases and hemicellulases. Their first aim is degradation of pectin, which results in loosening of cell wall structure and thereby enables digestion of the following polymers: cellulose and hemimcellulose [3, 4]. During the colonization in plants, antifungal compounds are generated: phytoalexins, PR proteins, small antifungal peptides and reactive oxygen species [5–7]. PR proteins are locally accumulated in the infection sites, in the adjacent tissues and also in non-infected tissues, which provides plant resistance to subsequent infections. Being part of the plant systemic response, these genes are not activated immediately after the pathogen attack, though their analysis was sufficient to establish the initial stage of the infection [8, 9].

The plant cell wall is a dynamic structure, composed of polysaccharide polymers (cellulose, hemicelluloses and pectin) and non-polysaccharide polymers (lignin) and proteins (structural and enzymatic) [10]. Cell wall composition is strictly regulated in the different types of cells during their growth, development and plant response to abiotic and biotic stress factors [11].

Cellulose consists of long, non-branched microfibrils composed of β-1,4-glucose chains, which are transversely connected with hydrogen bonds and van der Walls forces. There are two types of cellulose structure: highly polymerized and ordered and less polymerized, loose and amorphous [12]. The parameter that describes cellulose structure is the crystallinity index (CI), determining the content of crystalline form in the cellulose [13]. During cellulose biosynthesis, the key role is played by a large protein complex that is anchored in the cell membrane and consists of six subunits, each subunit consisting of six proteins (cellulose synthases; CESA) [14].

Hemicelluloses comprise heterogenic polysaccharides with low molecular mass. There are five different classes of hemicelluloses: xyloglucans, xylans, mannans, glucomannans and β-(1 → 3,1 → 4) glucans [15, 16]. Regarding the diversity of hemicellulose classes, many enzymes which belong to the protein family of glycosyltransferases are involved in the synthesis of this heterogenic polymer [17–19]. In the process of degradation of hemicelluloses many enzymes take part, *inter alia* endo-β-1,4-xylanase, exo-xylanase, β-1,4- xylosidase, α-1,4- xylosidase, α-arabinofuranosidase, α-glucuronidase, endo- β-mannase, β-mannosidase and β-glucosidase [18].

Pectin is a complex of polysaccharides, whose main constituents are molecules of galacturonic acid (GalAc) (around 70 % of all in pectin) bound with α-1,4-glycoside bonds. Additionally, there are rhamnose, arabinose, xylose, galacturonic acid and galactose. There are four structural types of pectin called pectin domains: homogalacturonan (HG), xylogalacturonan (XGA), rhamnogalacturonan I (RGI) and rhamnogalacturonan II (RGII) [20]. Around 70 enzymes, including glycosyltransferases, methyltransferases and acetyltransferases are involved in pectin biosynthesis. The most important are UDP-D-galacturonate 4-epimerase (GAE) [21], α-1,4-galacturonosyltransferase (GAUT), xylosyltransferase of rhamnogalacturonan II (RGXT) [22, 23], xylosyltransferase (XGD), arabinosyltransferase (ARAD), galactosyltransferase (GAL), xylosyltransferase [20, 21, 24], and methyltransferases (PMT). In the process of homogalacturonan hydrolysis, the following enzymes participate: exo- and endo-polygalacturonases (acting through hydrolysis), pectin lyase and pectate lyase (acting through transelimination), pectin methylesterases and pectin acetylesterases; in the hydrolysis of rhamnogalacturonan I: rhamnogalacturonan hydrolase, rhamnogalacturonan lyase, rhamnogalacturonan rhamnohydroxylase, rhamnogalacturonan rhamnohydrolase, rhamnogalacturonan galacturonhydroxylase and rhamnogalacturonan acetylesterase; and in the hydrolysis of xylogalacturonan: exo-polygalacturonase and endo-xylogalacturonan hydroxylase [25].

During pathogen infection pectin de-esterification plays a key role in the plant defence responses. Pectin de-esterification leads to the generation of free carboxyl groups, altering pH in the cell wall and enabling aggregation of polyuronates to the gel structure, which results in changes in the porosity of the cell wall [26]. Additionally, this process enables HG degradation by pectin polygalacturonases, pectin lyase and pectate lyase [27]. The level of methyl esterification of pectin determines the sensitivity of plants to the pathogen infection. The high content of methylated residues of galacturonic acid in HG corresponds to the increase of plant resistance [28, 29]. Moreover, the level and pattern of methyl esterification of pectin influence the activity of polygalacturonases, which are responsible for the generation of short fragments of homogalacturonan chains, which are oligogalacturonides (OG), endogenous molecules of elicitor activity that play a crucial role in the pathogen defence response by enhancing the plant natural response [30–32].

Lignin comprises a complex of aromatic polymers, which is mainly localized in the secondary cell wall of vascular plants. There are three types of lignin polymers: G lignins (guaiacyl-lignins), S lignins (syringyl lignin) and H lignins (hydroxy coumaryl lignin), which are composed of respective monolignols (hydroxycinnamic alcohols): coniferyl alcohol, synaptic alcohol and *p*-coumaric alcohol [33]. Lignification is a dynamic process consisting of generation of lignin polymers and their embedment in the plant cell wall [33]. This process consists of the following stages: monolignol biosynthesis in cytosol, transport of monolignols to the cell wall and polymerization in order to generate the lignin complex. The lignin synthesis pathway is a route of the phenylpropanoid pathway. Many enzymes participate in these reactions: phenylalanine ammonia lyase (PAL), 4-coumaric acid:coenzyme A ligase (4CL), hydroxycinnamoyl-CoA shikimate/quinate hydroxycinnamoyl transferase (HCT), caffeoyl-CoA O-methyltransferase (CCoAOMT), catechol-O-methyltransferase (COMT), cinnamoyl CoA reductase (CCR), synaptic alcohol reductase (SAD), cinnamyl alcohol dehydrogenase, cinnamic acid 4-hydroxylase, p-coumarate 3-hydroxylase and ferulate 5-hydroxylase (F5H) [34–36]. In the cell wall monolignols are activated by oxidation and generate stable monolignol radicals, which are able to bind to the growing lignin polymer. Reaction of monolignol oxidation is catalysed by peroxidases (POX), laccases (LAC) and other phenolic oxidase [33–35] enzymes which are also responsible for lignin degradation [37].

By strengthening the cell wall, lignin provides a better barrier to pathogen attacks. The increased lignin synthesis resulting from biotic stress factors results from phenylpropanoid pathway stimulation and from lignin polymerization [38, 39].

Development of genetic engineering enabled generation of genetically modified plants characterized by increased resistance to pathogen infections. Flax that was more resistant to *F. oxysporum* and *F. culmorum* infection was generated by overexpression of genes involved in pathogenesis (PR genes) [40] and genes of secondary metabolites [41–43].

It is justified to perform research on cell wall components in order to discover their significance for plant resistance to pathogens.

The aim of this study was to estimate the role of flax cell wall polymers in response to *Fusarium oxysporum*. The significance of cell wall polymers (cellulose, hemicelluloses, pectin and lignin) was elucidated by analysis of the expression level of genes implicated in the metabolism of these compounds and by the analysis of the corresponding metabolites in flax in response to pathogenic fungi.

## Results

### Phenotypic analysis of flax seedlings incubated with *Fusarium oxysporum*

In order to determine the role of cell wall polymers in flax in the response to a pathogenic strain of *Fusarium oxysporum* infected flax seedlings were incubated with the fungus for 6, 12, 24, 36 and 48 h. In the subsequent incubation period the transferred plants were photographed (Additional file 1: Figure S1). The first phenotypic changes of the flax seedlings were observed within 24 h after the transfer. Cotyledons of the seedlings remained green, while the adventitious root tips became necrotic and the necrosis progressed with the incubation time. Initially, after 24 h only a few root cells became necrotic, while after 48 h the necrotic changes were observed in most of the roots. Despite this, the cotyledons remained green and firm *F. oxysporum* mycelium was not observed on the surface of the MS medium. The last incubation period analyzed was 48 h after transfer, as at this stage the plants retained their green color and turgor, thus enabling activation of its defence mechanisms. In the consecutive hours of incubation the progress of the infection contributed to weakening and wilting of flax seedlings (data not shown); therefore their detailed analysis was abandoned.

### Expression of PR genes increased in flax seedlings infected with *Fusarium oxysporum*

**Fig. 1:**
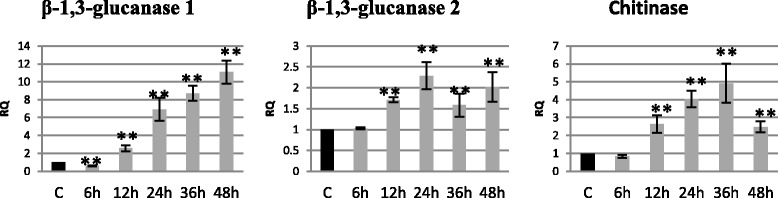
Relative expression of PR gene transcripts in flax seedlings infected with *Fusarium oxysporum*. Changes in expression levels of PR genes (β-glucanase 1, β-glucanase 2 and chitinase) in flax seedlings treated with pathogenic strains of *F. oxysporum* (F.ox.) at 6, 12, 24, 36 and 48 h after inoculation were presented as relative quantity (RQ) relative to reference gene (actin) in relation to control (C). The data were obtained from real-time RT-PCR analysis. The data represent the mean ± standard deviations from three independent experiments. The significance of the differences between the means was determined using Student’s *t* test (*P < 0.05, **P < 0.01)

In order to determine the earlier stages of infection we investigated the changes occurring during 6 and 12 h of incubation with the pathogen. We determined the levels of mRNAs of PR genes, because it is known that the genes are strongly expressed in plants in response to pathogen infections. Changes in PR gene expression in flax infected with a pathogenic *Fusarium oxysporum* fungus strain are presented in Fig. 1. The analyzed genes were characterized by an unchanged expression level in 6 h of incubation (β-1,3-glucanase 2 and chitinase) or with lower expression (by 40 %) followed by an increase in the subsequent hours of incubation in the case of β-1,3-glucanase 1. The level of expression of β-1,3-glucanase 1 increased during the period of incubation (from 2.6-fold in 12 h to 11-fold in 48 h). A similar expression pattern was found for the chitinase gene. The level of its transcript increased from 2.6-fold in 12 h to 4.9-fold in 36 h and dropped to 2.5-fold in the control. The analysis of β-1,3-glucanase 2 revealed the smallest changes in the expression in comparison to the other PR genes tested. However, compared to the control the level of mRNA of this gene increased 1.7-fold in 12 h, 2.3-fold in 24 h, 1.6-fold in 36 h and 2-fold in 48 h of incubation with *F. oxysporum*.

### Expression of cellulose metabolism genes changed in flax seedlings infected with *Fusarium oxysporum*

In the next step we analyzed changes in the levels of mRNAs of genes involved in cell wall polymer metabolism and determined their quantities in flax seedlings infected with a pathogenic strain of *F. oxysporum*. Polysaccharide (cellulose, hemicellulose, pectin) and non-polysaccharide (lignin) polymers were investigated.

**Fig. 2:**
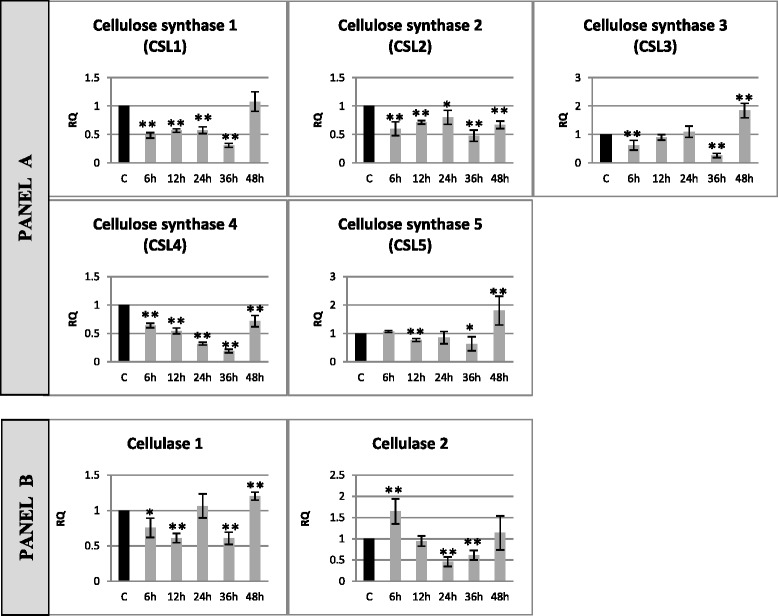
Relative expression of cellulose metabolism gene transcripts in flax seedlings infected with *Fusarium oxysporum*. Changes in expression levels of genes: cellulose synthesis (cellulose synthase isoform 1–5) – panel **a** and cellulose degradation (cellulase 1 and cellulase 2) – panel **b** in flax seedlings treated with pathogenic strains of *F. oxysporum* (F.ox.) at 6, 12, 24, 36 and 48 h after inoculation were presented as relative quantity (RQ) relative to reference gene (actin) in relation to control (C). The data were obtained from real-time RT-PCR analysis. The data represent the mean ± standard deviations from three independent experiments. The significance of the differences between the means was determined using Student’s *t* test (*P < 0.05, **P < 0.01)

Analysis of expression of genes of the synthesis and degradation of cellulose (5 isoforms of cellulose synthase and 2 isoforms of cellulase) in flax incubated for 48 h with a pathogenic *F. oxysporum* strain is presented in Fig. 2. Cellulose synthesis genes were characterized by a twofold expression pattern. In the first group (CSL1, CSL2 and CSL4) the expression levels were lowered (from 20 to 80 % depending on the gene analyzed and incubation time), while in the second group (CSL3 and CSL5) the expression was first reduced (to 60 % in 6 h and 26 % in 36 h for CSL3 and to 77 % in 12 h and 64 % in 36 h for CSL5) to increase 1.8-fold for both genes (CSL3 and CSL5). Among the levels of mRNAs of cellulose degradation genes, the expression of the cellulase 1 gene decreased (from 75 % in 6 h to 61 % in 12 and 36 h) and then increased 1.2-fold in 48 h of incubation with *F. oxysporum*. The expression of cellulase 2 initially increased 1.65-fold (in 6 h) and then decreased (to 46 % in 24 h and 60 % in 36 h).

### Cellulose content increases in flax seedlings infected with *Fusarium oxysporum*

**Fig. 3:**
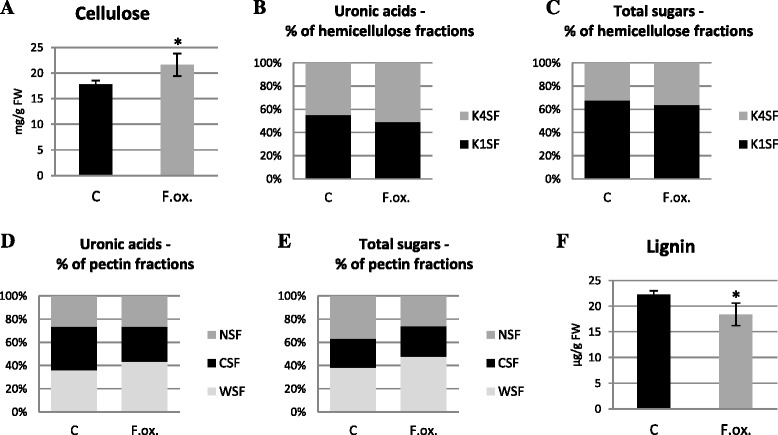
Content of cell wall polymers in flax seedlings infected with *Fusarium oxysporum*. Changes in cellulose (**a**) and lignin (**f**) amount as well as the content of uronic acids and monosaccharides in hemicellulose (**b** and **c**) and pectin (**d** and **e**) in flax seedlings treated with pathogenic strains of *F. oxysporum* (F.ox.) at 48 h after inoculation relative to control flax (C) were determined by spectrophotometric methods. K1SF – 1 M KOH soluble fraction; K4SF – 4 M KOH soluble fraction; WSF – water soluble fraction; CSF – CDTA soluble fraction; NSF – Na_2_CO_3_ soluble fraction. Data represent the mean ± SD from four independent measurements. The significance of the differences between the means was determined using Student’s *t* test (*- *P* < 0.05, **- *P* < 0.01)

Analysis of cellulose content revealed a 20 % increase in flax seedlings incubated for 48 h with *Fusarium oxysporum* compared to non-infected seedlings (Fig. 3a). The amounts of cellulose did not change in the remaining hours of incubation of flax with the pathogen.

### Expression of hemicellulose metabolism genes changed in flax seedlings infected with *Fusarium oxysporum*

**Fig. 4:**
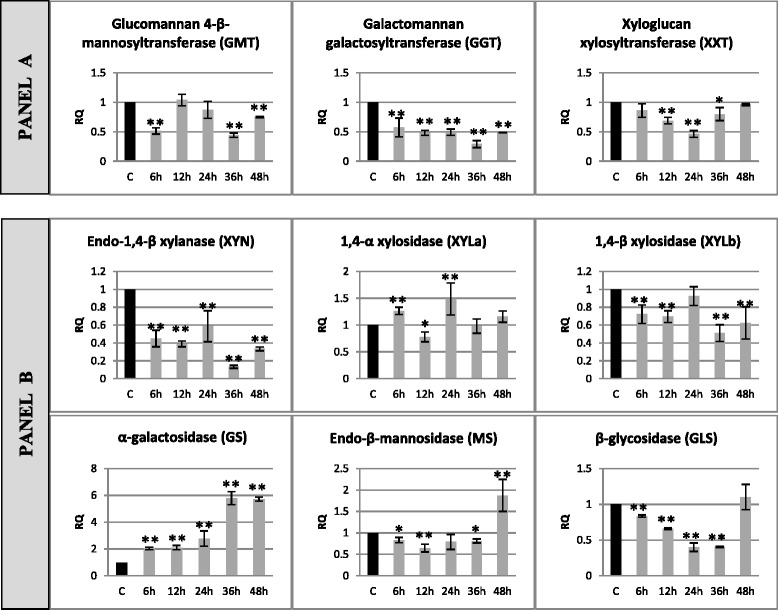
Relative expression of hemicellulose metabolism gene transcripts in flax seedlings infected with *Fusarium oxysporum*. Changes in expression levels of genes: hemicellulose synthesis (glucomannan 4-β-mannosyltransferase – GMT, galactomannan galactosyltransferase – GGT, xyloglucan xylosyltransferase – XXT) – panel **a** and degradation (endo-1,4-β-xylanase – XYN, 1,4-α-xylosidase – XYLa, 1,4-β-xylosidase – XYLb, α-galactosidase – GS, endo-β-mannosidase – MS, β-glycosidase – GLS) – panel **b** in flax seedlings treated with pathogenic strains of *F. oxysporum* (F.ox.) at 6, 12, 24, 36 and 48 h after inoculation were presented as relative quantity (RQ) relative to reference gene (actin) in relation to control (C). The data were obtained from real-time RT-PCR analysis. The data represent the mean ± standard deviations from three independent experiments. The significance of the differences between the means was determined using Student’s *t* test (*P < 0.05, **P < 0.01)

Results depicting changes in the levels of expression of genes involved in hemicellulose synthesis (glucomannan 4-β-mannosyltransferase – GMT, galactomannan galactosyltransferase – GGT, xyloglucan xylosyltransferase – XXT) and degradation (endo-1,4-β-xylanase – XYN, 1,4-α-xylosidase – XYLa, 1,4-β-xylosidase – XYLb, α-galactosidase – GS, endo-β-mannosidase – MS, β-glycosidase – GLS) in flax incubated with a pathogenic strain of *F. oxysporum* are presented in Fig. 4.

The expression of tested genes of hemicellulose synthesis decreased. The decrease (ranging from 57 to 29 %) of GGT gene expression was observed during the whole time of incubation of flax with the pathogen (6–48 h), while reduction of GMT gene expression was noted in 6 h (to 50 %), in 36 h (to 44 %) and in 48 h (to 75 %) and in XXT gene expression in 12, 24 and 36 h, to 70, 46 and 80 %, respectively. A decrease in expression level was also observed in some of the genes participating in the process of hemicellulose degradation. The level of expression of the XYN gene was lowered in all analyzed incubation periods of the incubation of flax with *F. oxysporum* (from 40 % in 24 h to 87 % in 36 h). A decrease in the expression level was also noted for XYLb and GS, and for XYLb it was constant (about 30 % in 6, 12, 36 and 48 h), while for GS the reduction in the expression intensified during the incubation (a 17 % decrease in 6 h and 60 % in 36 h). The MS gene was characterized by an initial decrease in the expression by 20–35 % in 6, 12 and 36 h and 1.9-fold increase in 48 h. Another pattern of expression after incubation with *F. oxysporum* was observed for the XYLa gene, whose expression increased 1.3-fold in 6 h, in 12 h it decreased to 78 %, and in 24 h it increased again 1.5-fold. The last among the analyzed genes of hemicellulose degradation, GS, was characterized by an increased level of expression ranging from 2-fold in 6 h to 5.8-fold in 36 and 48 h of incubation of flax with *F. oxysporum*.

### Hemicellulose composition changed in flax seedlings infected with *Fusarium oxysporum*

Hemicellulose contents were characterized by total simple sugar and total uronic acid contents in different hemicellulose fractions of cell wall (K1SF – 1 M KOH soluble fraction; K4SF – 4 M KOH soluble fraction) in flax seedlings infected with *F. oxysporum* for 48 h. Total uronic acid (Additional file 2: Figure S2A) and total simple sugar (Additional file 2: Figure S2B) contents remained unchanged in the seedlings after *F. oxysporum* infection and the content was 1 mg/g FW and 17 mg/g FW, respectively. Changes in uronic acid (Fig. 3b) and simple sugar (Fig. 3c) contents were observed in particular hemicellulose fractions. Uronic acid content decreased in the K1SF fraction and increased in K4SF after the infection. A similar association was noted for simple sugars. In addition, analysis of the simple sugars in both fractions indicated an increased contribution of the K1SF fraction to total hemicellulose.

### Expression of pectin metabolism genes changed in flax seedlings infected with *Fusarium oxysporum*

**Fig. 5:**
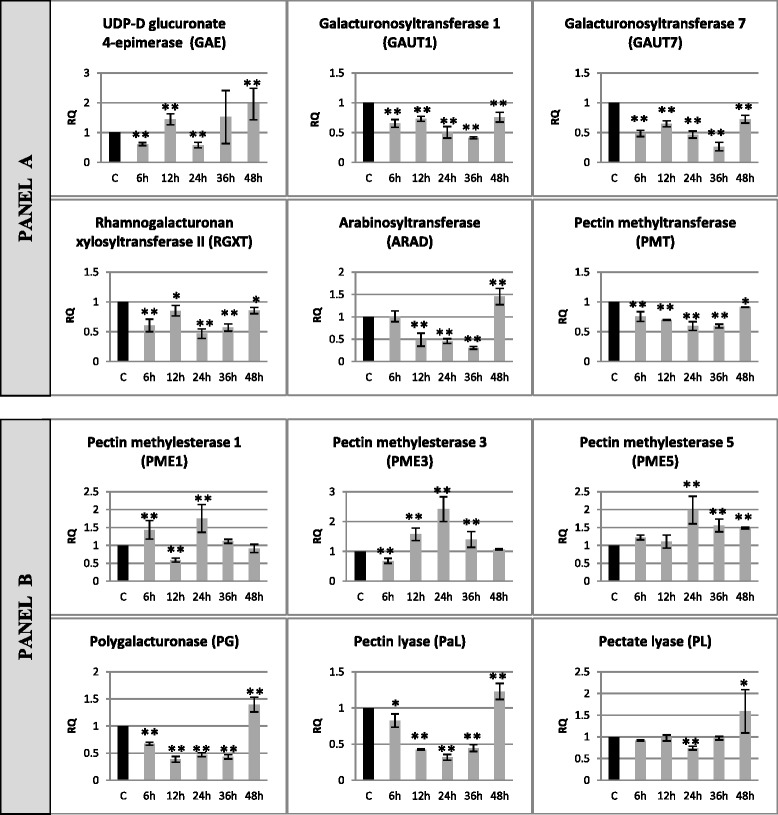
Relative expression of pectin metabolism gene transcripts in flax seedlings infected with *Fusarium oxysporum*. Changes in expression levels of genes: pectin synthesis (UDP-glucuronate 4-epimerase – GAE, galacturonosyltransferase 1 – GAUT1, galacturonosyltransferase 7 – GAUT7, rhamnogalacturonan II xylosyltransferase – RGXT, arabinose transferase – ARAD, pectin methyltransferase – PMT) – panel **a** and degradation (pectin methylesterase 1 – PME1, pectin methylesterase 3 – PME3, pectin methylesterase 5 – PME5, polygalacturonase – PG, pectin lyase – PaL, pectate lyase – PL) – panel **b** in flax seedlings treated with pathogenic strains of *F. oxysporum* (F.ox.) at 6, 12, 24, 36 and 48 h after inoculation were presented as relative quantity (RQ) relative to reference gene (actin) in relation to control (C). The data were obtained from real-time RT-PCR analysis. The data represent the mean ± standard deviations from three independent experiments. The significance of the differences between the means was determined using Student’s *t* test (*P < 0.05, **P < 0.01)

Changes in expression levels of the genes of pectin synthesis (UDP-glucuronate 4-epimerase – GAE, galacturonosyltransferase 1 – GAUT1, galacturonosyltransferase 7 – GAUT7, rhamnogalacturonan II xylosyltransferase – RGXT, arabinose transferase – ARAD, pectin methyltransferase – PMT) and degradation (pectin methylesterase 1 – PME1, pectin methylesterase 3 – PME3, pectin methylesterase 5 – PME5, polygalacturonase – PG, pectin lyase – PaL, pectate lyase – PL) in flax incubated with a pathogenic strain of *F. oxysporum* for 48 h (in consecutive hours of incubations: 6, 12, 24, 36, 48 h) are presented in Fig. 5.

In the majority of the analyzed genes of pectin synthesis (GAUT1, GAUT7, RGXT and PMT) a decrease in their expression was observed (by 15–70 %) upon pathogen treatment in all the incubation times. Expression of the ARAD gene was reduced to 50–30 % in 12, 24 and 36 h, but in 48 h it increased 1.4-fold compared to the control. The GAE gene was characterized by a variable pattern of expression of which in 6 and 24 h a 40 % decrease, while in 12 and 48 h a 1.44-fold and 2-fold increases was noted. Expression of pectin methylesterases changed over the time of incubation with *F. oxysporum* and in 24 h a 1.8-fold increase for PME1, 2.4-fold increase for PME3 and 2-fold increase for PME5 were observed. Moreover, the expression of PME5 increased 1.5-fold in 36 and 48 h, the expression of PME1 increased 1.4-fold in 6 h, but fell to 60 % in 12 h and the expression of PME3 decreased to 68 % in 6 h and increased 1.5-fold in 12 and 36 h. Expression of PG, PaL and PL genes initially decreased (67–43 % for PG and 82–32 % for PaL from 6 to 36 h and to 75 % for PL in 24 h) and then increased 1.4-fold for PG, 1.23-fold for PaL and 1.6-fold for PL in 48 h of incubation.

### Pectin composition changed in flax seedlings infected with *Fusarium oxysporum*

Pectin content was evaluated based on the analysis of uronic acid and simple sugar contents. Because uronic acids are the main structural components, the pectin assay is often based on the analysis of these constituents. In order to perform detailed evaluation of pectin content, all simple sugars in consecutive pectin fractions of cell wall (WSF – water soluble fraction, CSF – CDTA soluble fraction, NSF – Na_2_CO_3_ soluble fraction) must be assayed. Uronic acid content should be analyzed and need not be omitted because of partial qualitative analysis of pectin.

Total uronic acid content (about 5 mg/g FW) and simple sugars (about 12 mg/g FW) did not change in flax incubated for 48 h (Additional file 2: Figure S2C and D). However, uronic acid contents in particular pectin fractions of cell wall differ, indicating their higher content in the CSF fraction in the control seedlings (37.5 % of total pectin) and in the WSF fraction in the seedlings infected with *F. oxysporum* (43.6 % of total pectin) (Fig. 3e). The content of uronic acids in the NSF fraction did not change after the infection (26 % of total pectin). Differences were observed in simple sugars in pectin fractions between the infected and control flax seedlings (Fig. 3e). After infection with *F. oxysporum* the content of simple sugars in the WSF fraction increased by 10 % compared to the control, but did not change in the CSF fraction and decreased in the NSF fraction.

### Lignin metabolism gene expression increased in flax seedlings infected with *Fusarium oxysporum*

**Fig. 6:**
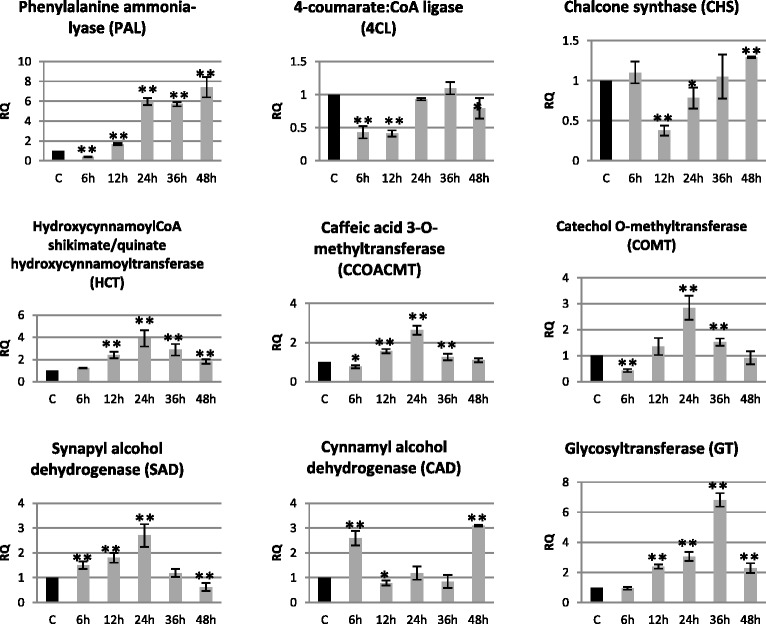
Relative expression of selected genes of phenylpropanoid pathway transcripts in flax seedlings infected with *Fusarium oxysporum*. Changes in expression levels of phenylpropanoid metabolism genes: phenylalanine ammonia lyase – PAL, 4-hydroxycinnamoyl : CoA ligase – 4CL, chalcone synthase – CHS, p-hydroxycinnamoyl CoA : quinic/shikimic acid transferase – HCT, caffeoyl-CoA O-methyltransferase – CCoAOMT, caffeic acid/5-hydroxyferulic acid 3/5-O-methyltransferase – COMT, synaptic acid dehydrogenase – SAD, hydroxycinnamic alcohol dehydrogenase – CAD, glucosyltransferase – GT in flax seedlings treated pathogenic strains of *F. oxysporum* (F.ox.) at 6, 12, 24, 36 and 48 h after inoculation were presented as relative quantity (RQ) relative to reference gene (actin) in relation to control (C). The data were obtained from real-time RT-PCR analysis. The data represent the mean ± standard deviations from three independent experiments. The significance of the differences between the means was determined using Student’s *t* test (*P < 0.05, **P < 0.01)

Analysis of lignin metabolism gene expression (phenylalanine ammonia lyase – PAL, 4-hydroxycinnamoyl : CoA ligase – 4CL, chalcone synthase – CHS, p-hydroxycinnamoyl CoA : quinic/shikimic acid transferase – HCT, caffeoyl-CoA O-methyltransferase – CCoAOMT, caffeic acid/5-hydroxyferulic acid 3/5-O-methyltransferase – COMT, synaptic acid dehydrogenase – SAD, hydroxycinnamic alcohol dehydrogenase – CAD, glucosyltransferase – GT) in flax seedlings incubated for 48 h with a pathogenic strain of *F. oxysporum* is presented in Fig. 6.

Among all the analyzed genes, four (HCT, CCoAOMT, COMT and SAD) showed the same expression pattern during the incubation with *F. oxysporum* compared to the control. Their expression increased from 6 h of the incubation to reach the maximum in 24 h (3.9-fold increase in HCT expression and 2.7-fold increase in expression of CCoAOMT, COMT and SAD) and decreased in 48 h to a level equal to the control (CCoAOMT, COMT), a level below the control (40 % lower expression of SAD) and a level above the control (HCT expression level of 1.8-fold of the control). Similarly, although shifted in time, the pattern of expression was characteristic for the GT gene, whose expression reached a maximum in 36 h (6.8-fold). PAL gene expression was initially lowered to 40 % in 6 h, and increased gradually to reach a maximum in 48 h (7.4-fold increase compared to the control). 4CL gene expression was decreased to 40 % in 6 and 12 h and to 80 % in 48 h. Analysis of the last of the genes of lignin metabolism (CAD) showed a 2.6- and 3.1-fold increase in mRNA level in 6 and 48 h, respectively, and a 30 % decrease in 12 h of incubation with *F. oxysporum*.

### Lignin content decreases in flax seedlings infected with *Fusarium oxysporum*

Lignin content was assayed in flax seedlings incubated with *F. oxysporum* for 48 h, and the results are presented in Fig. 3f. The infection caused a decrease of lignin content in flax seedlings by about 20 % in comparison to the non-infected seedlings.

### Infrared spectroscopy of cell wall of flax infected with *Fusarium oxysporum* confirms results of biochemical analysis of cell wall components

Analysis of infra-red spectroscopy of the cell wall of flax infected with *F. oxysporum* was performed to determine the structure of the cell wall and verify the results of cell wall polymer content assay obtained with spectrophotometric methods.

**Fig. 7:**
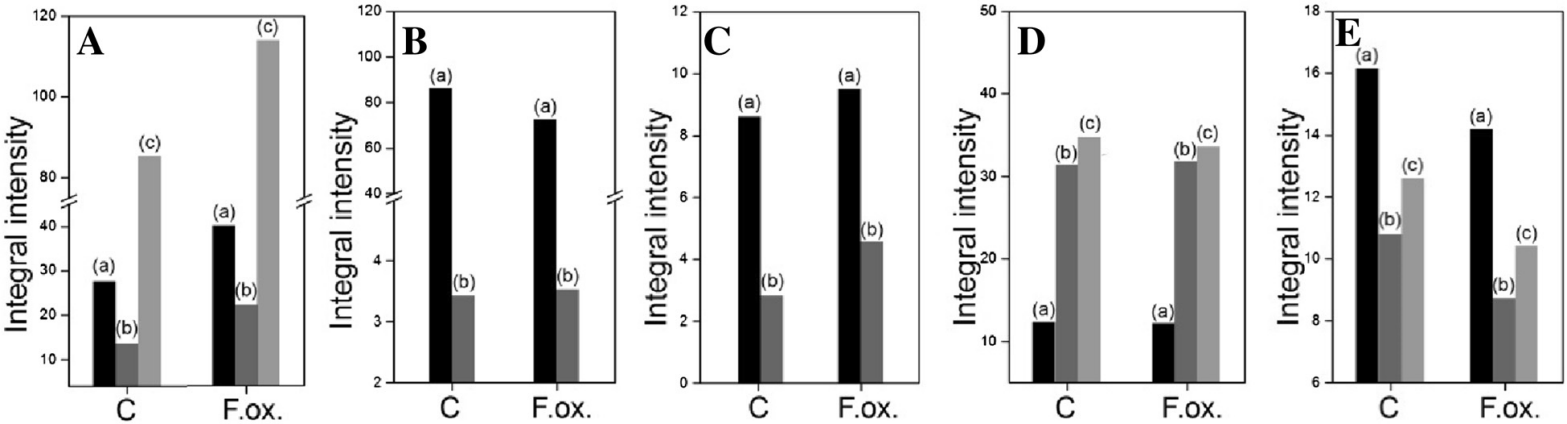
IR spectrophotometry analysis of the cell wall structure and composition of flax seedlings infected with *Fusarium oxysporum*. The IR spectra of samples from control flax seedlings (C), seedlings after *F. oxysporum* infection (F.ox.). **a** Changes in cellulose content presented as differences in the integral intensities of the bands at 1455 cm^−1^ (a), 1319 cm^−1^ (b), and 1161 cm^−1^ (c). **b** Changes in the structure of cellulose (C-O-C bonds) presented as differences in the integral intensities of the bands at 1058 cm^−1^ (a) and 988 cm^−1^ (b). **c** Changes in cellulose structure presented as differences in the integral intensities of the bands at 1230 cm^−1^, corresponding to δ (OH · · · O) (a) and 625 cm-1, corresponding to γ(OH•••O) (b). **d** Changes in pectin content presented as differences in the integral intensities of the bands at 1735 cm^−1^ (a), 1655 cm^−1^ (b) and 1609 cm^−1^ (c). **e** Changes in lignin content presented as differences in the integral intensities of the bands at 1337 cm^−1^ (a), 1260 cm^−1^ (b) and 1245 cm^−1^ (c)

The infection of flax seedlings with *F. oxysporum* influenced the composition and structure of the cell wall. Infrared spectroscopy spectra of the infected and non-infected flax seedlings are presented in Fig. 7. Based on the spectra changes in the cellulose, pectin and lignin contents and changes in cellulose structure were determined in the studied samples. The cellulose content was 40 % higher after the infection with *F. oxysporum* compared to the non-infected seedlings (Fig. 7a). Cellulose structure was determined based on the analysis of appropriate bands. Integral intensities of bands at 1058 and 988 cm^−1^ corresponding to asymmetric vibrations of ν(C-O-C) indicate changes in the length of cellulose chains, which were shorter in the infected flax seedlings (Fig. 7b). Integral intensities of bands in the range of 3400–3425 cm^−1^ correspond to intra-molecular vibrations O-H × × × O and in the range of 3290–3315 cm^−1^ correspond to inter-molecular vibrations ν(O-H × × × O) (Fig. 7c). The analyzed results show changes in the arrangement of cellulose chains in flax seedlings after infection with *F. oxysporum* and a 60 % higher number of hydrogen bonds compared to the non-infected seedlings. Infected flax seedlings display increased the crystallinity index by 16 %, indicating a more organized cellulose structure in the cell wall of the infected flax seedlings. Higher crystallinity also suggests lower reactivity of cellulose, lower water absorption and higher plasticity of cell walls.

Changes in pectin and lignin contents determined by the analysis of differences in the integral intensities of bands at 1735, 1655 and 1609 cm^−1^ for pectin (Fig. 7d) and at 1337, 1260 and 1245 cm^-1^ for lignin (Fig. 7e) confirm a significant decrease of pectin and lignin contents after infection with *F. oxysporum*.

## Discussion

Nowadays the research on host-pathogen interaction is of great interest to enable amelioration of plant defence mechanisms.

The current literature describes only the importance of pectin during plant infection and omits other polysaccharide polymers of the cell wall. The research aim of this study was to determine the significance of polysaccharide polymers and lignin during different stages of infection of a pathogenic strain of *Fusarium oxysporum*. It was suggested to approach this whole scientific problem and pay attention to different polymers and not to omit the possible interaction in the described process.

In order to examine the different stages of flax infection by *F. oxysporum*, flax seedlings incubated with the pathogen were collected after 6, 12, 24, 36 and 48 h. The choice of incubation period was established experimentally based on the phenotype of infected plants, determining the last incubation time and the PR gene expression whose level significantly increased in plants infected with pathogens [44–46]. Expression of genes of β-1,3-glucanase and chitinase in flax incubated with *F. oxysporum* were induced after 12 h, and their level increased in time. The results indicated that in the 12 h of incubation in spite of the lack of phenotype changes, the pathogen infected the plant and induced a systemic response. In order to examine the first stage of infection comprising pathogen penetration to the root cells and activation of the defence mechanism by the host, the 6th hour of incubation was chosen as the first hour.

The first analyzed polymer in flax infected with *F. oxysporum* was pectin, the main aim for pathogen in the initial stages of infection. Pectin methylesterases remove the methyl group from homogalacturonan, resulting in loosening of cell wall structures, enabling pectin degradation by polygalacturonases and pectin lyases, and also cellulose and hemicellulose by cellulases and hemicellulases [47]. It is then suggested that during the first stage of infection the methylation level of pectin plays the main role. Highly methylated pectin causes higher resistance to plant infection. In plants, there are endogenous pectin methylesterases that take part in many physiological processes, in which rearrangement of the cell wall is necessary.

Analysis of three isoforms of pectin methylesterases showed that during the first stage of infection expression of PME1 increased, PME3 decreased and PME5 did not change. These results indicate that PME5 probably does not take part in the infection process, but is involved in other physiological processes, although its level increased after 24 h of incubation. PME1 is involved in infection, and the alteration of the expression level of PME1 is putatively caused by a pathogen. A previous study confirmed that infecting fungi can influence the increase in PME gene expression [48], and silencing of a specific PME gene, which depends on the pathogen, led to an increase in plant resistance [49]. Nevertheless, the initial increase in PME1 expression can be explained by the purposeful plant strategy which aims to loosen the structure of pectin to release oligogalacturonides (OG) in order to elicit a plant defence response. Transgenic strawberries with overexpression of one of the isoforms of pectin methylesterase were characterized by increased resistance to *Botrytis cinerea* infection, which was explained by the release of oligogalacturonides from the cell wall [50]. Summing up, the observed decreases and increases in expression of PME1 and PME3 genes are probably caused by plant defence mechanisms in response to the ongoing infection: initially by maintaining high pectin methylation and then by its rearrangement and/or release of elicitors (OG).

Biochemical analysis of pectin in seedlings after flax infection with *F. oxysporum* confirmed its rearrangement, which was observed after 48 h in spite of no changes in the total content. Possibly, the activity of endogenous pectin methylesterases of fungi and endogenous pectin methylesterases of flax induced by the pathogen lead during the progressing infection to demethylation of pectin, and then to loosening of its structures by exogenous polygalacturonases and pectin lyases. Pectin rearrangement revealed an increase in the water-soluble fraction and lower content in the CDTA fraction with a simultaneous lack of change in its content in the fraction bound with the cell wall. Loosening of pectin structure is a result of breaking ion bonds by the activity of fungal enzymes [51].

Lowered mRNA levels of genes of polygalacturonase and pectin lyase from the first stage of infection (6 h) until the moment when the cell necrosis of roots is more advanced results from the presence of exogenous fungal genes (PG and PaL), which comprise a form of defence against excessive pectin degradation. In contrast, the decrease in the expression level of most genes of pectin synthesis during the whole infection process might result from redirection of transcription to other genes, which are necessary in abundance during infection. Loosening and rearrangement of pectin made accessible other polymers of the cell wall to exogenous fungal enzymes: hemicellulases and cellulases. Pathogens degrade polysaccharides in order to ensure themselves the nutrients, mainly glucose. Hemicellulose is responsible for the strengthening of the cell wall [52], but until now no participation in the plant response to pathogen infection has been documented. *Fusarium oxysporum* caused a reduction of the expression level of genes of hemicellulose synthesis at the first stage of infection of flax seedlings. Similarly, most genes of hemicellulose degradation were observed to exhibit a lowered expression level. Such changes might be explained by the altered transcription profile in plants that undergo pathogen infection. Probably, plants redirect their transcription potential to other genes necessary during pathogen-derived stress. Despite the lowering of expression of genes of hemicelluloses metabolism, their content did not change, and only slight lowering of the hemicellulose fraction soluble in 1 M KOH was observed.

Analysis of gene expression of cellulose metabolism in flax seedlings infected with *F. oxysporum* revealed a decrease in mRNA level of three isoforms of cellulose synthesis genes (CSL1, CSL2 and CSL4) in all studied incubation times. Two other isoforms of cellulose synthase and two isoforms of cellulose degradation genes responded to infection weakly or unspecifically. A decrease in expression of genes of cellulose synthesis was previously described only in *Arabidopsis thaliana* infected with *Botrytis cinerea* and revealed the involvement of genes of this polymer in the response to pathogen infection. Such a phenomenon was explained by modulation of transcription, but the hypothesis has not been confirmed yet [53]. A previous study showed that *Arabidopsis thaliana* with silenced cellulose synthase genes is characterized by increased resistance to *Ralstonia solanacearum* and *Plectosphaerella cucumerina* infections [54]. Regarding the difficulties in explaining the role of cellulose synthases in plant resistance to pathogens, the content and structure of cellulose was studied. The quantitative spectrophotometric analysis revealed an increase in cellulose content in seedlings infected with *F. oxysporum* for 48 h. Similarly, infrared spectroscopic analysis of the cell wall of infected seedlings showed a significant increase of cellulose with a 60 % increase of hydrogen bonds in cellulose and a 16 % increase of cellulose crystallinity. Increased crystallinity of cellulose means a more orderly structure, which is characterized by lower activity, lower water absorbance and increased plasticity of the cell wall. It is then deduced that the increase of cellulose crystallinity is an additional barrier of plants generated during infection, which impedes host cell wall polymer digestion by pathogen enzymes.

Alteration of cellulose crystallinity in the cell wall of seedlings infected with the pathogen is difficult to explain. It is suggested that it was due to rearrangements of hemicelluloses. Despite the lack of expression of most genes of hemicellulose metabolism, one of them exhibited strong activation during infection. The mRNA level of α-galactosidase increased at the first stage of infection, and then was increasing during the period of infection. Putatively, this enzyme that cuts off galactose from galactomannan indirectly enabled generation of hydrogen bonds in cellulose and thereby led to the increase of its crystallinity. This hypothesis needs further research and confirmation. According to other authors [55], enzymes that degrade hemicelluloses in the cell wall and release galactose can directly contribute to the alteration in cellulose crystallinity.

Lignin’s involvement in pathogen infections is well known and described in the literature. Increased lignin biosynthesis results from stimulation of the phenylpropanoid pathway, while the lignification process is induced by reactive oxygen species, present in great amounts during pathogen infection [38, 39]. Increase in expression of genes of the phenylpropanoid pathway and direct genes of lignin synthesis was observed in wheat infected with *Fusarium graminearum* and *Puccinia triticina* [55] and in cotton infected with *Verticillium dahliae* [56].

Expression of genes of the phenylpropanoid pathway (PAL, HCT, CCoAOMT, COMT and GT) was strongly induced during the later stages of seedling infection with *F. oxysporum* and increased during the time of infection. These changes were observed after 12 h of incubation, similarly in the case of PR genes, suggesting their involvement in the systemic flax response. At the initial stage of infection of *F. oxysporum* (after 6 h), genes of lignin synthesis – hydroxycinnamic alcohol dehydrogenase and synaptic alcohol dehydrogenase –were characterized by a significant increase of mRNA level. The results suggest that lignin synthesis occurs quite fast in order to strengthen the cell wall, but may be limited by the accessibility of substrates. Biochemical analysis and IR spectrometry of the cell wall of flax seedlings after 48 h of infection with *F. oxysporum* revealed a slight decrease in lignin content. Perhaps this short time from the infection onset is not sufficient to show alteration in this polymer. In cotton infected by pathogenic fungi, despite the significant increase of expression level of genes of lignin metabolism, from 12 h, changes in lignin content were observed after several days following infection onset [56].

Summing up, analysis of the expression level of genes of polymer cell wall metabolism in flax infected with *F. oxysporum* enabled us to distinguish two group of genes that responding differently to infection. The first group consisted of strongly reacting genes, whose expression levels were increased over 5–10 times: genes of lignin synthesis (phenylalanine ammonia lyase [PAL], glucosyltransferase [GT]) and genes involved in pathogenesis (β-1,3-glucanase and chitinase). They were characterized as systemic response genes, because despite the significant expression level increase, genes responded later (after 12 h of incubation with pathogens), and the maximum expression increase was mostly at 48 h. The second group of genes comprised genes slightly induced. They participate in the metabolic response, and their activation is connected with activity of elicitors (oligogalacturonides) generated as a result of cell wall digestion by fungal enzymes. In this group there are genes whose expression level increased (up to five times), decreased, and genes with different, often unspecific expression alterations. The first subgroup consisted of other lignin synthesis genes, the β-glycosidase gene (GLS), and β-1,3-glucanase 2. In the second subgroup of genes with lowered expression level were genes of pectin metabolism (GAUT1, GAUT7, RGXT, PMT, PME3, PG and PaL), hemicellulose metabolism (GMT, GGT, XXT, XYN, XYLb and GS) and cellulose synthesis (cellulose synthases: CSL1, CSL2 and CSL4). The last subgroup, which exhibited different changes in expression, consists of other genes including pectin, hemicellulose and cellulose metabolism. It is suggested that genes that respond weakly to pathogen infection are only transcriptional noise and they do not take part in plant defence mechanisms.

## Conclusion

The results of this study confirm that polymers of the cell wall participate in the flax response to *Fusarium oxysporum* infection through the changes in expression of their genes and rearrangement of cell wall structure*.* Although the role of pectin and lignin has been widely studied and the literature provides much information, our studies confirmed the contribution of pectin and lignin to the infection process through the loosening of the pectin structure and the increase at mRNA levels of genes participating in lignin synthesis. We provided new information about the role in the pathogen infection of cellulose and hemicellulose, which are expressed by changes in their structure rather than their content, as well as in the profile of their transcriptome. Our preliminary studies encourage further research to precisely determine the mechanism of participation of cellulose and hemicelluloses in the infection process.

## Methods

### Plant material preparation

All experiments were performed on a fibrous flax variety (*Linum usitatissimum* L. cv. Nike). The pathogenic strain *Fusarium oxysporum* f. sp. *linii* (Bolley) Snyder et Hansen (ATCC MYA-1201) was purchased from the ATCC Company (USA). The plants were grown on MS medium solidified with agar (0.8 %) supplemented with 1 % sucrose in a plant growth chamber under the following conditions: humidity: 50 %, temperature 22 °C/16 °C, light intensity: 23 mmol/s/m^3^, day/night regime: 16/8 h.

Seed germination and seedling development proceeded under controlled conditions. After 7 days the seedlings were transferred (with the medium) onto PDA medium overgrown with a pathogenic strain of *Fusarium oxysporum* or control PDA (without the fungus). The fungus was grown on the PDA medium for 5 days prior to the experiment. The seedlings (100 for each stage) were collected after 6, 12, 24, 36 and 48 h, frozen in liquid nitrogen and stored at −70 °C.

### Gene expression analysis

For total RNA isolation TRIzol reagent (Life Technologies, USA) was used. The isolation was performed according to the producer’s protocol in triplicate. In order to purify the isolated RNA from the remaining DNA, a DNaseI kit (Invitrogen, Germany) was used according to the manufacturer’s protocol. For reverse transcription reaction, that is cDNA synthesis on an RNA matrix, the High Capacity cDNA Reverse Transcription Kit (Life Technologies, USA) was used according to the manufacturer’s instructions.

Real-time PCR was performed in a StepOnePlus Real-Time PCR Systems thermocycler (Applied Biosystems, USA) using the DyNAmo SYBR Green qPCR Kit (Thermo Scientific, USA), according to the manufacturer’s protocol. Primers were prepared to not amplify fungal sequences. Their annealing temperature was 57 °C and their sequences are presented in Additional file 3: Table S1. Real-time PCR reactions were performed in three repetitions for each of the analyzed samples. Changes in gene expression levels were calculated as relative quantities (RQ) of the reference gene (actin) and presented as x-fold of gene expression in relation to non-treated plants.

### Determination of cellulose content

Cellulose content was determined with the anthrone method described by Ververis [57]. Tissue samples (100 mg) were incubated with a mixture of 65 % nitric acid and 80 % acetic acid (1:8 v/v) for 1 h at 100 °C and then centrifuged for 5 min at 14 000 rpm. The pellet was then washed twice with water and dissolved in 1 ml of 67 % H_2_SO_4_ (v/v) by shaking at room temperature for 1 h. 100 μl of extract was added to 900 μl of cooled solution of 0.2 % anthrone in 67 % sulfuric acid, mixed, heated at 100 °C for 15 min and cooled down on ice. Cellulose content in the sample was determined by spectrophotometric measurement of absorbance at 620 nm. Commercially available cellulose was used for standard curve preparation.

### Isolation and fractionation of cell wall polysaccharides

Isolation and fractionation of cell wall constituents were performed using modified methods described by Manganaris and Vicente [51, 58]. Flax tissue was incubated with 96 % ethanol for 30 min at 100 °C in order to inactivate enzymes, to extract components of low molecular mass and to prevent autolysis. Next, the pellet was incubated with the following: 80 % of ethanol for 20 min at 80 °C, chloroform:methanol mixture (1:1 v/v) for 1 h at 40 °C and acetone for 5 min. The samples were centrifuged (for 5 min at 5000 x g) between each solvent treatments and the supernatant was discarded. The pellet was dried overnight at 37 °C and its constituents insoluble in alcohol (AIR) were used for further analysis.

The pellet (AIR) was dissolved in 1 ml of water and was shaken for 12 h at room temperature, centrifuged (6000 × g, 4 °C, 10 min) and rinsed with water. Supernatants derived from two centrifugations were combined and used for analysis as a water soluble fraction (WSF). The following fractions of cell wall were obtained by the same procedures, but water was substituted with other solvents: CSF (fraction soluble in CDTA) – 50 mM CDTA (1,2-cyclohexylenedinitrilotetraacetic acid) pH 6.5; NSF (fraction soluble in sodium carbonate) – 50 mM Na_2_CO_3_ with 20 mM NaBH_4_, additionally, neutralization with acetic acid of collected supernatants; K1SF (fraction soluble in 1 M KOH) – 1 M KOH with 20 mM NaBH_4_, neutralized with hydrochloric acid; K4SF (fraction soluble in 4 M KOH) – 4 M KOH, neutralization with hydrochloric acid. Supernatants of fractions CSF, NSF, K1SF and K4SF were dialyzed to water (membranes for dialysis 3.5-kDa), and all fractions were lyophilized before use for further analysis.

### Determination of uronic acid content

The content of uronic acids was determined with the biphenol method [59] with the prior hydrolysis of polysaccharides with sulfuric acid [60]. Lyophilized samples (around 10 mg) were dissolved in 0.1 ml of concentrated sulfuric acid in a cooling bath with shaking for 5 min. Then, 0.1 ml of sulfuric acid, 0.05 ml of water, 0.05 ml of water and 0.7 ml of water were added with shaking between each portion. After the centrifugation (10 min, 2000 x g, RT), 0.1 ml of the supernatant was taken, and 10 μl of 4 M aminosulfonic acid of pH 1.6 was added and 600 μl of 75 mM sodium tetraborate in concentrated sulfuric acid was added. After careful mixing, the samples were incubated for 20 min at 100 °C, cooled down and 20 μl of *m*-hydroxybiphenyl (0.15 %) in 0.5 % NaOH was added, and they were left at room temperature for 10 min. The content of uronic acids was measured spectrophotometrically at 525 nm. As a standard for the calibration curve, glucuronic acid was used.

### Determination of total monosaccharide content with phenolic method

Content of total monosaccharide was determined with the phenolic method with prior hydrolysis with sulfuric acid. To 0.3 ml of the supernatant, 0.6 ml of concentrated sulfuric acid was added, mixed, and 50 μl of 5 % phenol in water was added. The samples were incubated for 20 min at 50 °C, and after cooling down, the level of monosaccharides was measured spectrophotometrically at 480 nm. As a standard for the calibration curve, glucuronic acid was used.

### Lignin content determination

The total lignin content was determined with the acetyl bromide method described by Iiyama and Wallis [61]. One hundred milligrams of flax seedlings were heated for 2 h at 100 °C and then 10 ml of water was added and heated for 1 h at 65 °C, shaking every 10 min. Samples were filtered through GF/A 24 mm filters, and were three times rinsed with the following: water, ethanol, acetone, diethyl ether. Filters were placed in glass vials and were heated overnight at 70 °C. Then, 2.5 ml 25 % (*v/v*) of acetyl bromide in 80 % acetic acid was added and incubated for 2 h at 50 °C. The cooled down samples were mixed with 10 ml of 2 M sodium hydroxide and 12 ml of acetic acid. After the overnight incubation the lignin content was determined spectrophotometrically at 280 nm. As a standard for the calibration curve, coniferyl alcohol was used.

### Infra-red spectroscopy analysis of flax cell walls

IR spectrometry was used to determine the chemical composition and molecular structure of cell walls from the flax seedlings. The spectra were measured at room temperature using a Bio-Rad 575C FT-IR spectrometer. Data were collected over a spectral range from 50 to 4000 cm^−1^ with a resolution of 2 cm^−1^. In the mid infra-red part of this range, samples were prepared in a KBr pellet. In the far infra-red part of this range, samples were suspended in Nujol. The crystallinity index (CI), which describes structural organisation, in this case cellulose, was calculated as the intensity ratio of the bands at 1370 cm^−1^ (−CH vibration) and 2900 cm^−1^ (−CH2- and -CH vibrations).

### Statistical analysis

All experiments were independently repeated at least three times. Obtained results were presented as the mean values ± standard deviation. Statistical analysis was performed using Statistica 7 (StatSoft, USA). To determine the statistical significance, Student’s *t*-test (*P < 0.05, **P < 0.01) was used.

## Additional files

Additional file 1: Figure S1. Phenotypic analysis of flax seedlings inoculated with pathogenic strain of *Fusarium oxysporum*. Phenotypic changes in flax seedlings infected with *F. oxysporum* at 6, 12, 24, 36 and 48 h after inoculation in comparison with non-infected control plants. The vertical panels show the phenotype of flax seedlings in the incubation period, and the horizontal panels show: control seedlings, magnified control seedlings, flax seedlings infected with *F. oxysporum* and magnified infected seedlings, respectively. (DOCX 18171 kb) Additional file 2: Figure S2. The content of total uronic acids and total monosaccharides in hemicellulose and pectin fraction in flax seedlings infected with *Fusarium oxysporum*. Changes in total uronic acids (Additional file 2: Figure S2A and C) and total monosaccharaides (Additional file 2: Figure S2B and D) amount of hemicellulose and pectin fraction estimated based on the amount of uronic acids (Fig. 3 b and d) and monosaccharides (Fig. 3c and e). K1SF – 1 M KOH soluble fraction; K4SF – 4 M KOH soluble fraction; WSF – water soluble fraction; CSF –CDTA soluble fraction; NSF –Na_2_CO_3_ soluble fraction. Data represent the mean ± SD from four independent measurements. The significance of the differences between the means was determined using Student’s *t* test (*- *P* < 0.05, **- *P* < 0.01). (DOC 955 kb) Additional file 3: Table S1. Primer sequences for real-time RT-PCR reactions. Primer sequences designed for real-time PCR: (A) Cellulose metabolism genes. (B) Hemicellulose metabolism genes (C) Pectin metabolism genes. (D) Lignin metabolism genes. (E) PR genes and actin. (DOCX 20 kb)
