# Supplementary material for: Evaluation of the significance of cell wall polymers in flax infected with a pathogenic strain of Fusarium oxysporum
Source: BMC Plant Biol. 2016 Mar 22;16:75. doi: 10.1186/s12870-016-0762-z (PMC4804541; doi:10.1186/s12870-016-0762-z)
Supplement: Additional file 1: Figure S1. — Phenotypic analysis of flax seedlings inoculated with pathogenic strain of Fusarium oxysporum. Phenotypic changes in flax seedlings infected with F. oxysporum at 6, 12, 24, 36 and 48 h after inoculation in comparison with non-infected control plants. The vertical panels show the phenotype of flax seedlings in the incubation period, and the horizontal panels show: control seedlings, magnified control seedlings, flax seedlings infected with F. oxysporum and magnified infected seedlings, respectively. (DOCX 18171 kb) [file 12870_2016_762_MOESM1_ESM.docx]

| **CONTROL** | **CONTROL** | ***FUSARIUM OXYSPORUM*** | ***FUSARIUM***  ***OXYSPORUM*** |
| --- | --- | --- | --- |
| 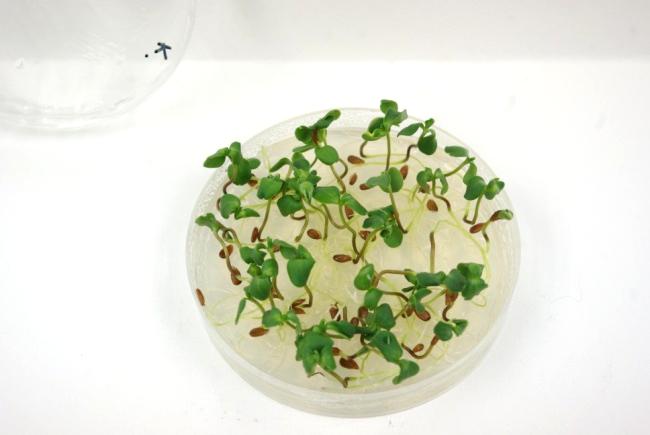  **6 HOURS OF THE INCUBATION** | 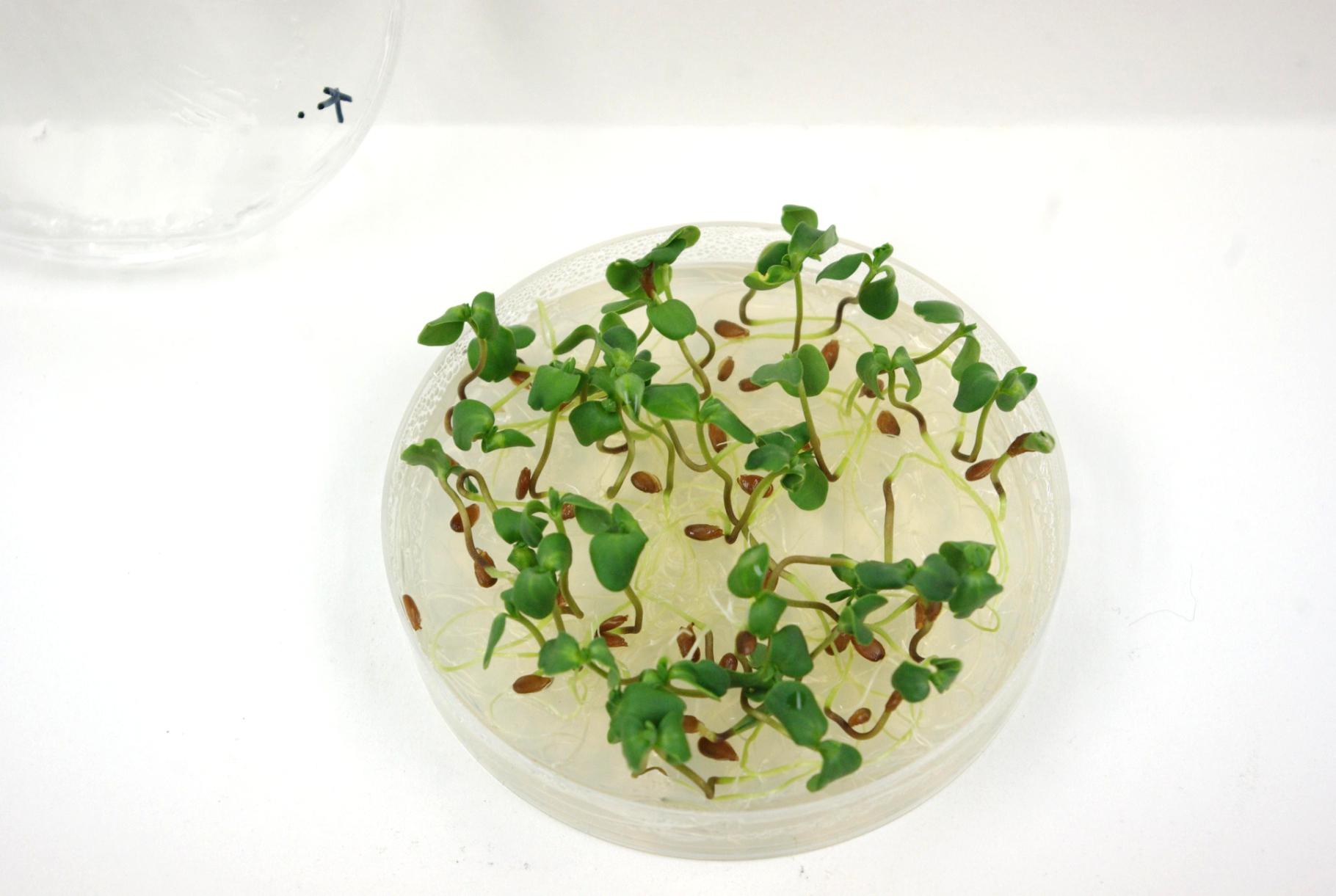 | 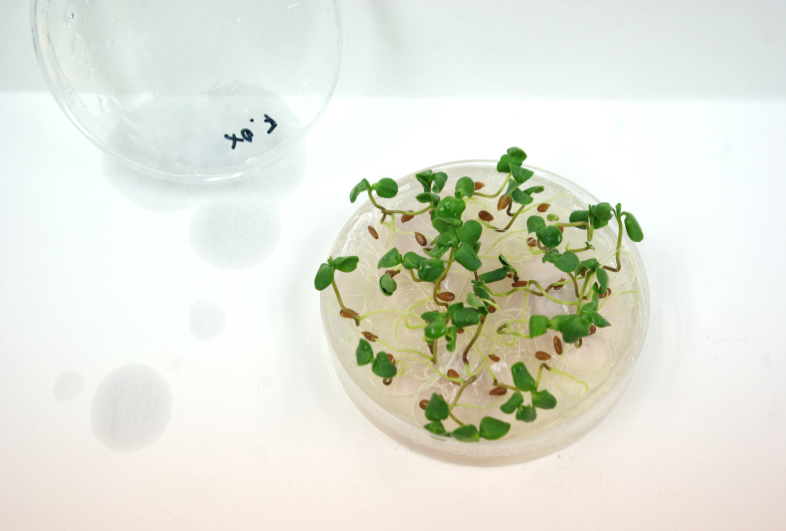 | 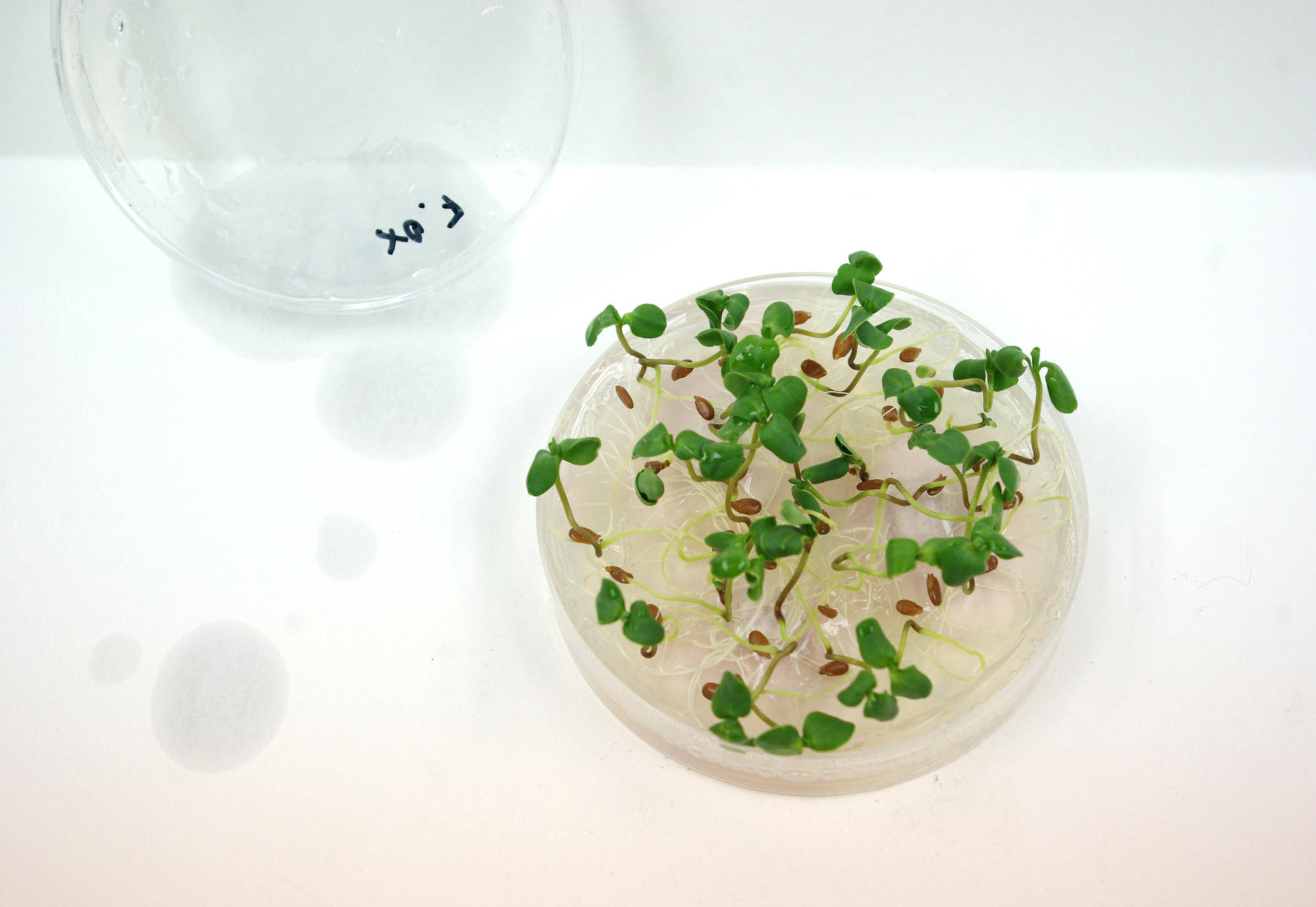 |
| 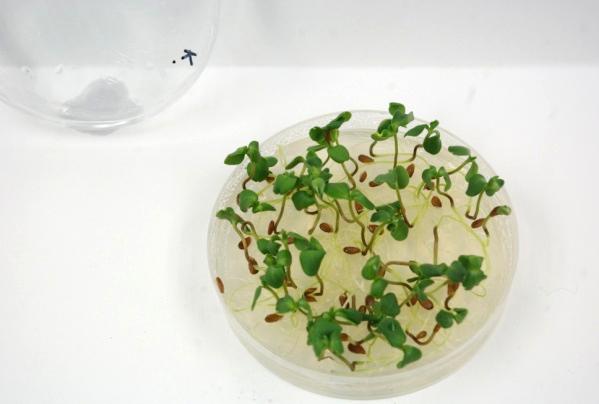  **12 HOURS OF THE INCUBATION** | 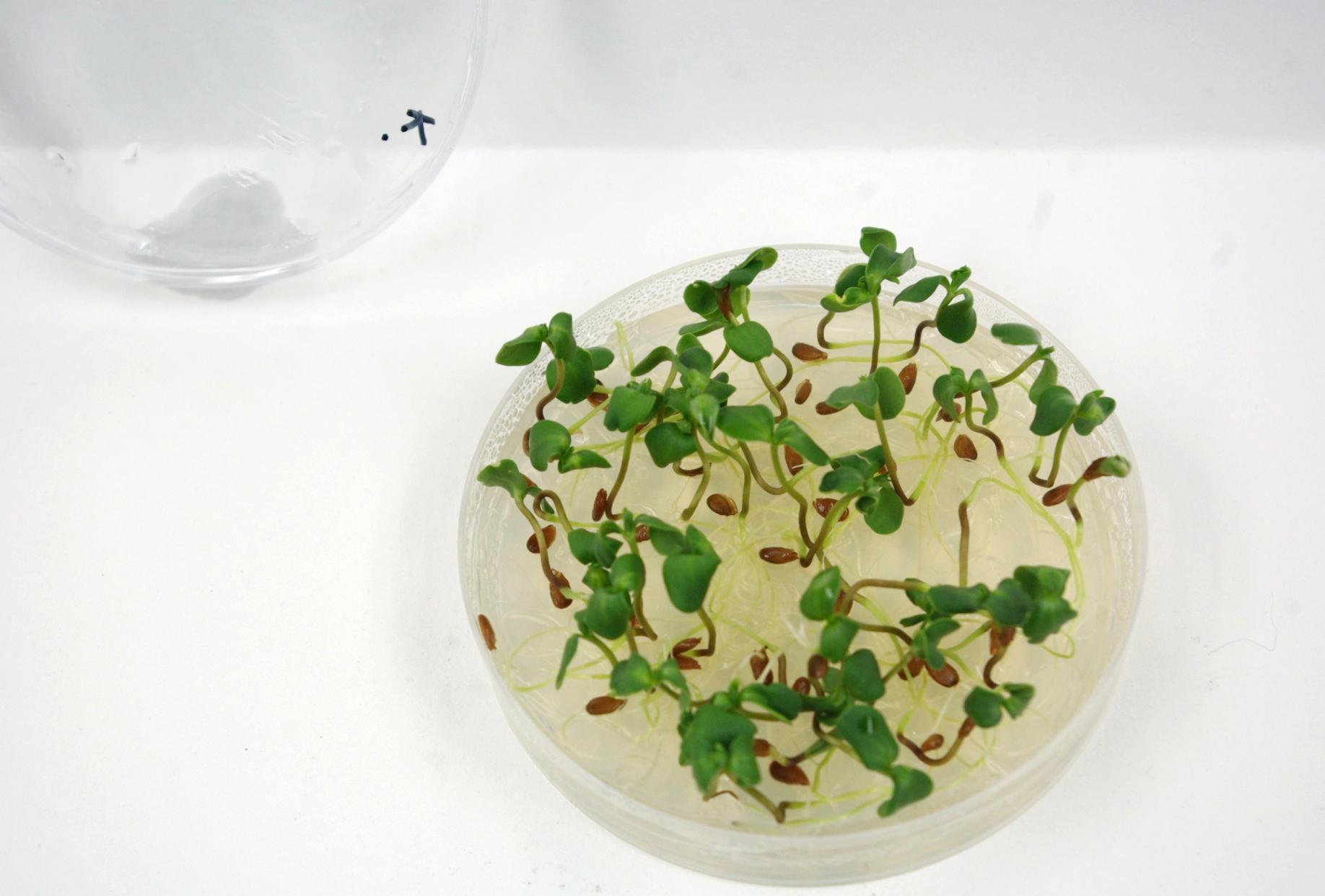 | 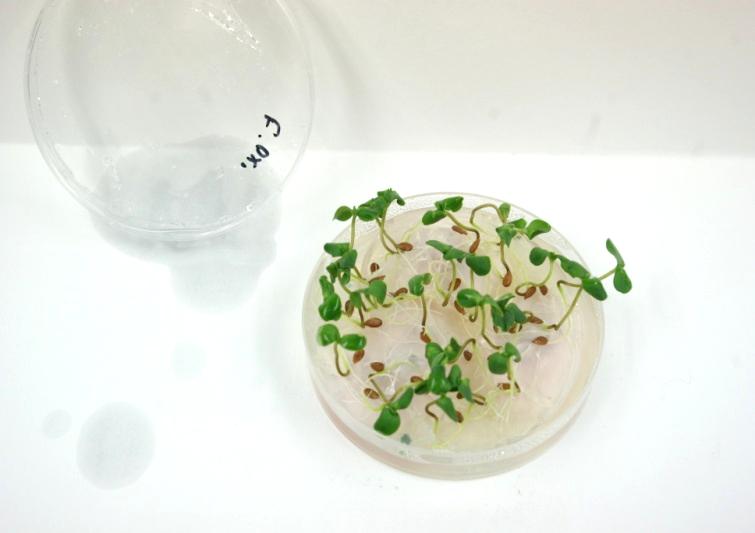 | 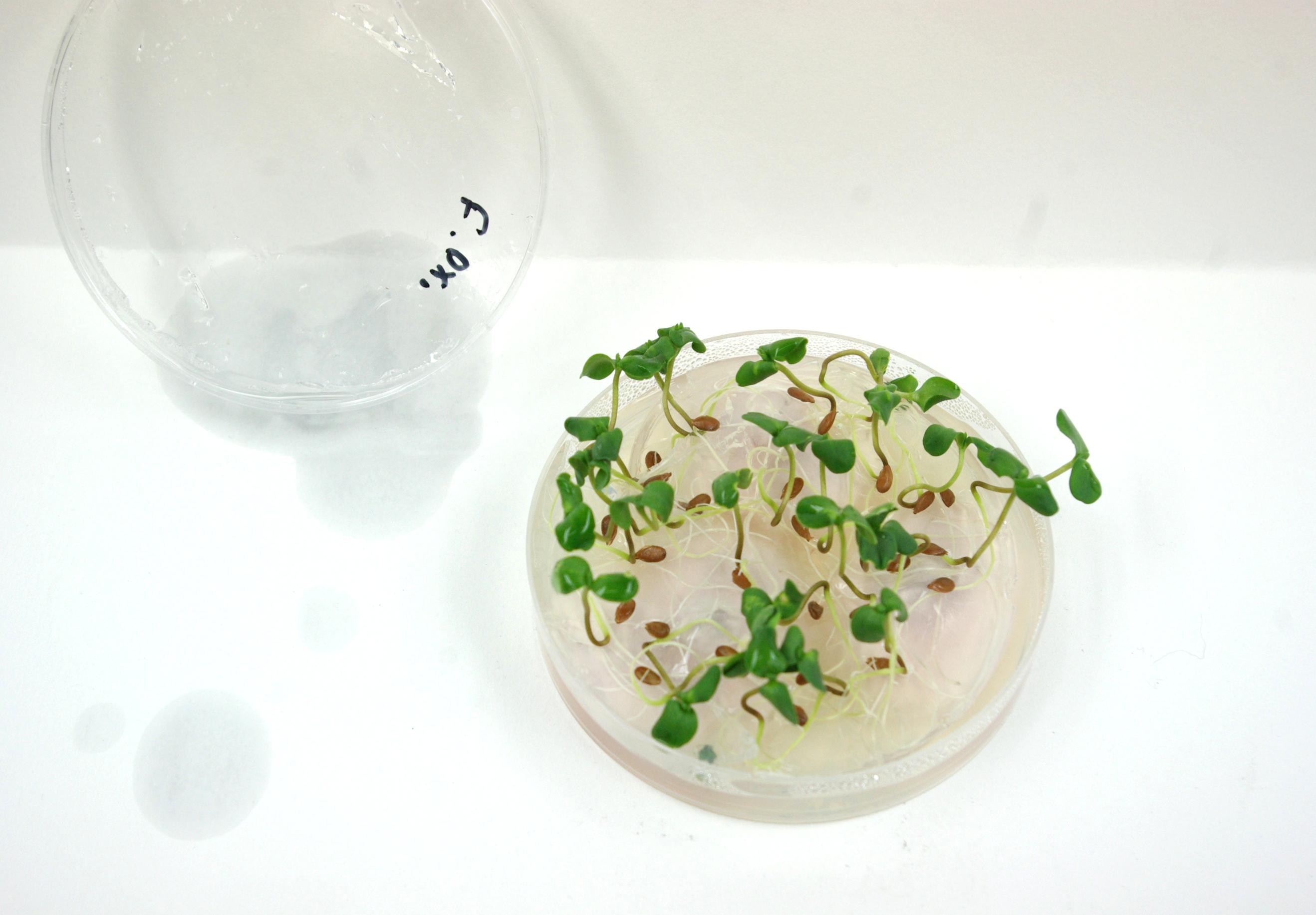 |
| 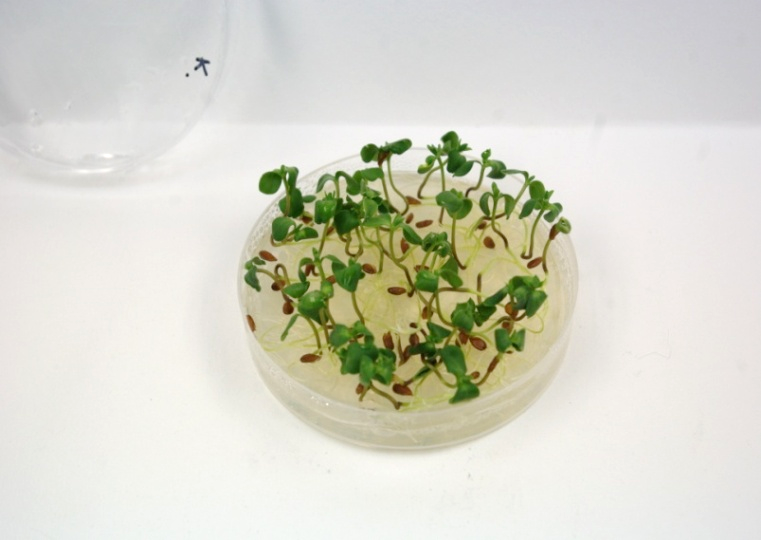  **24 HOURS OF THE INCUBATION** | 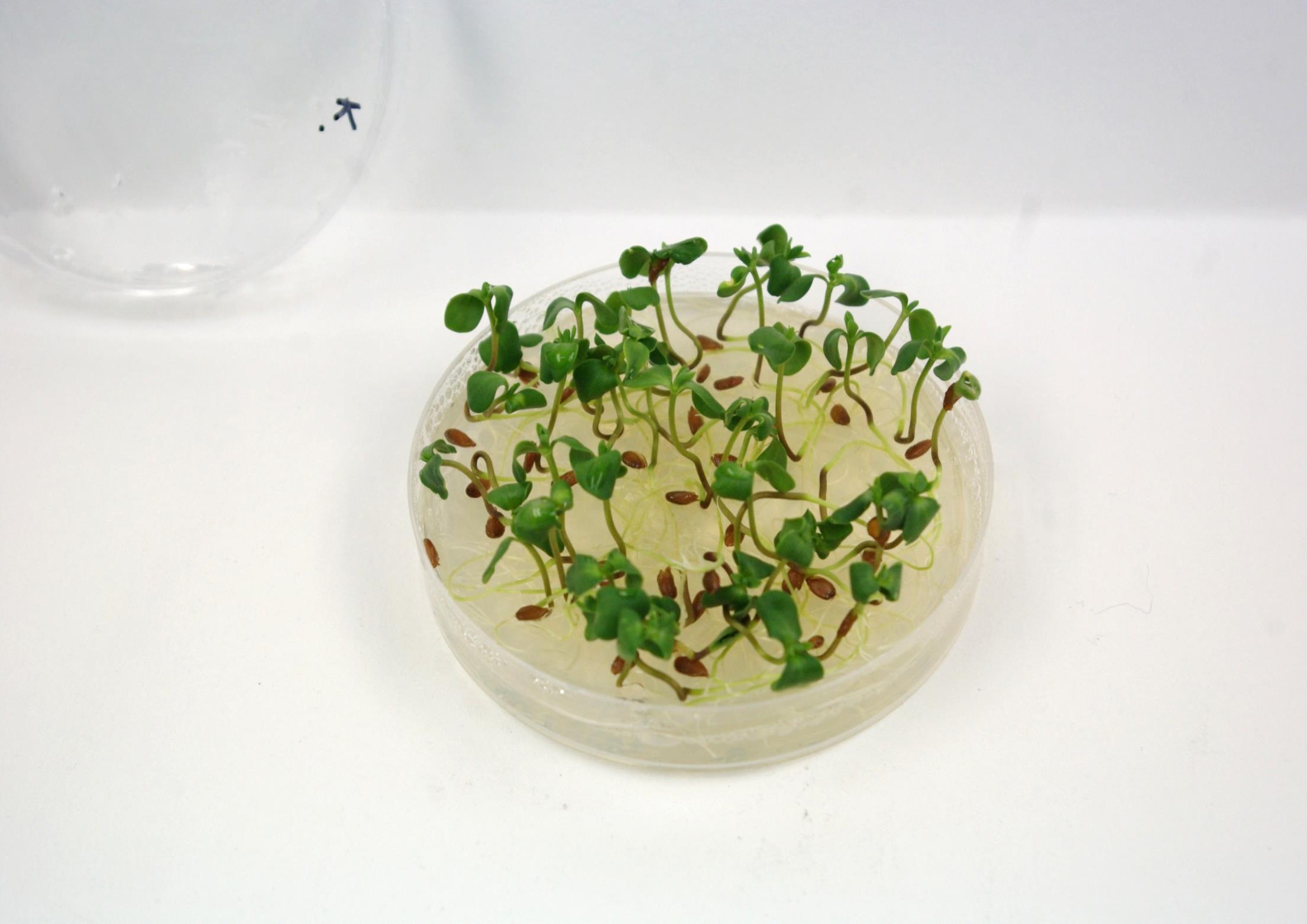 | 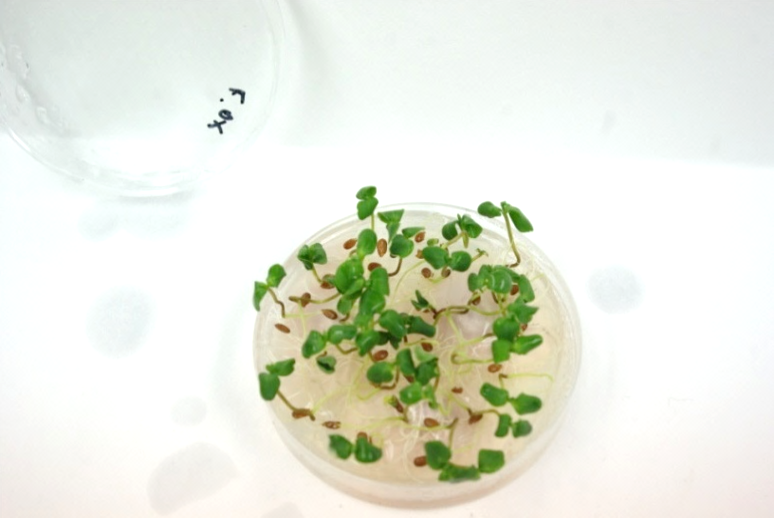 | 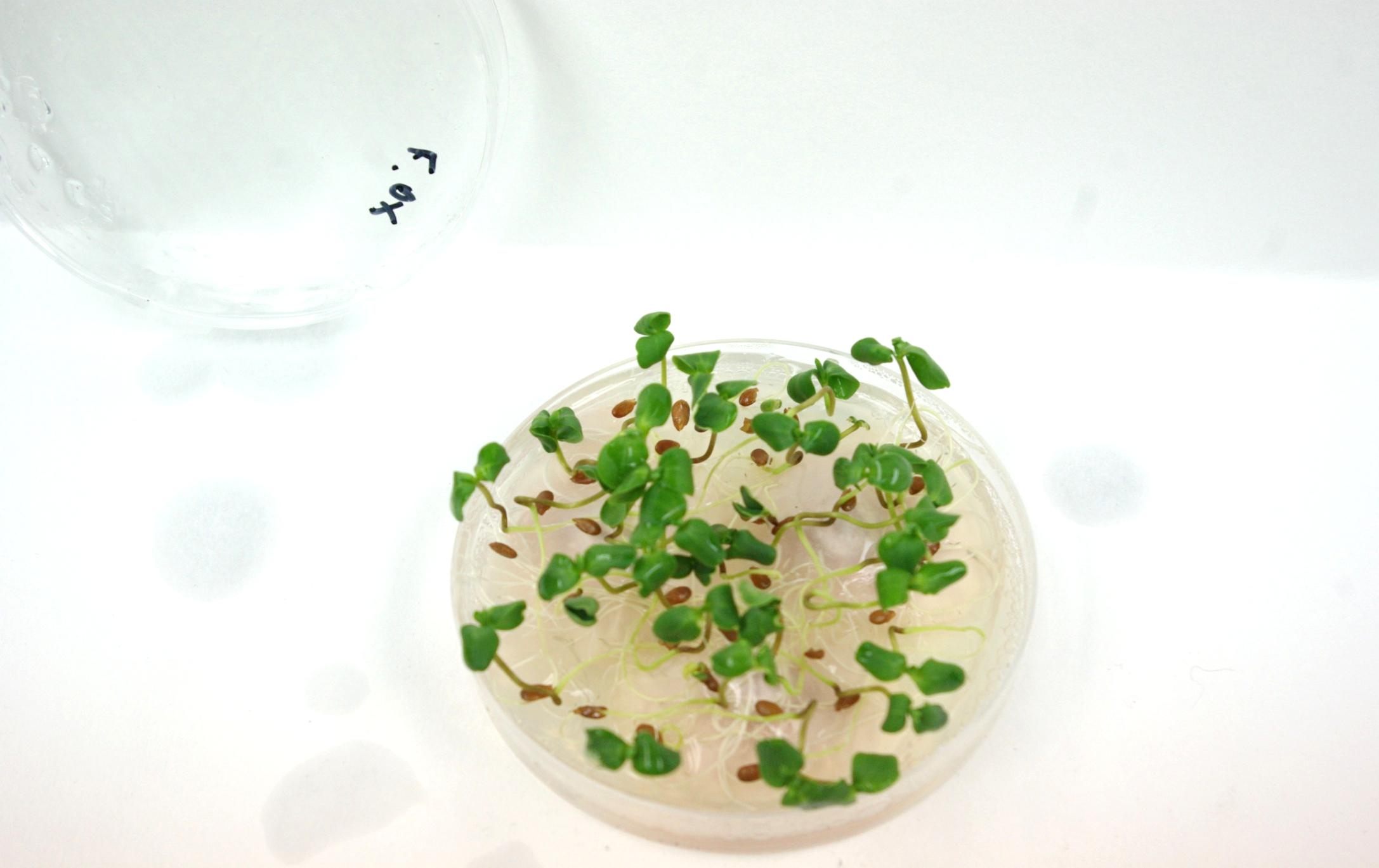 |
| 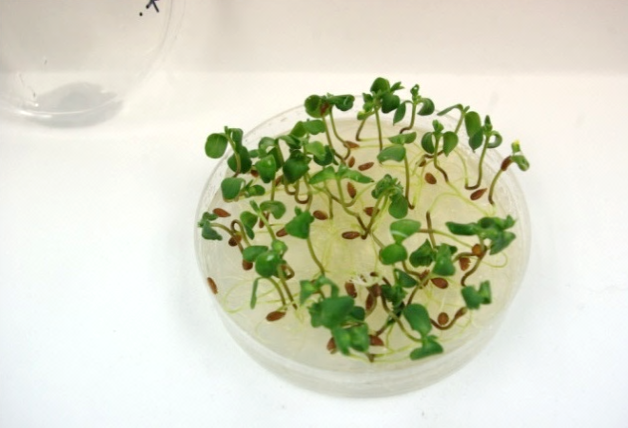  **36 HOURS OF THE INCUBATION** | 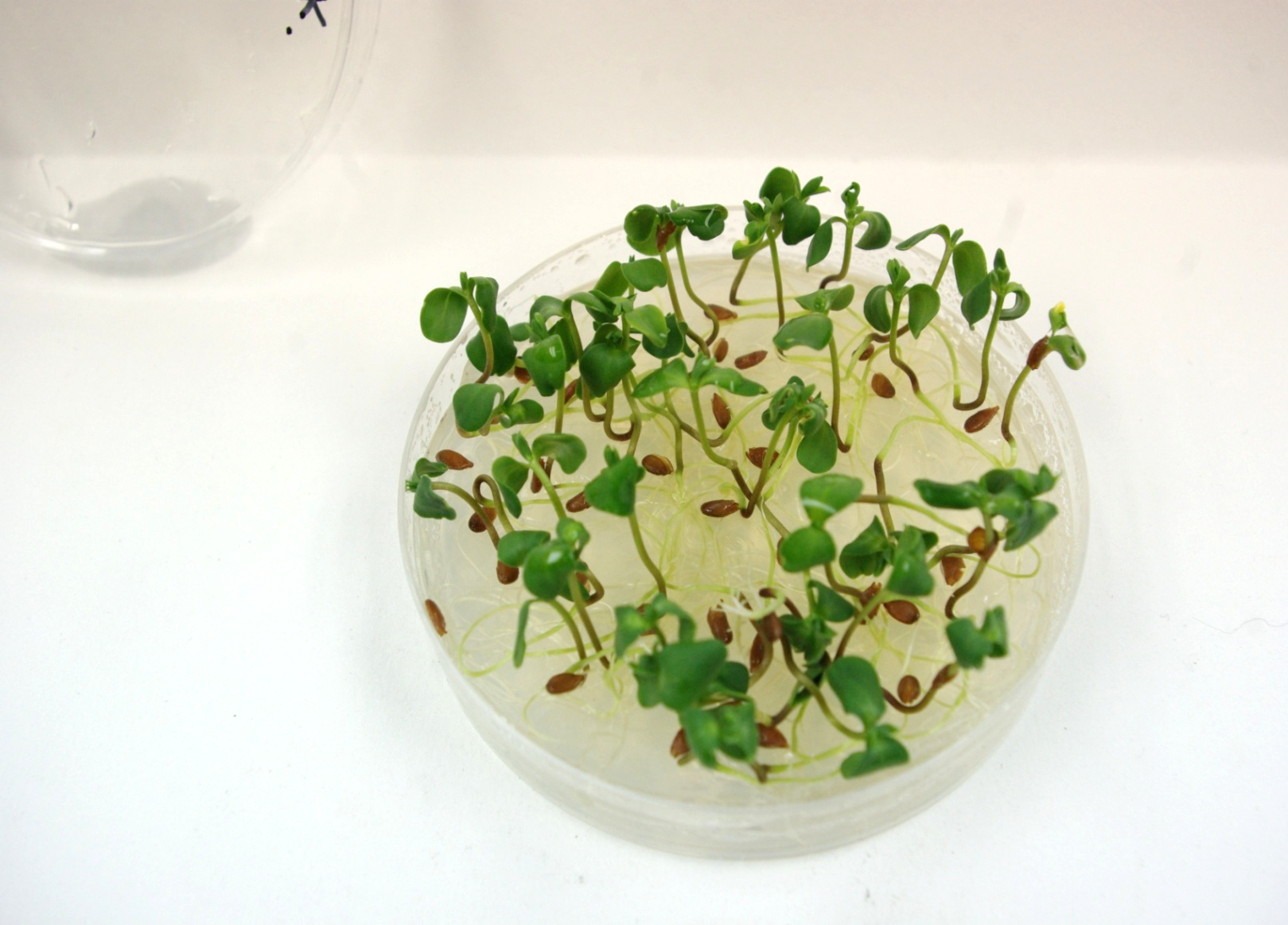 | 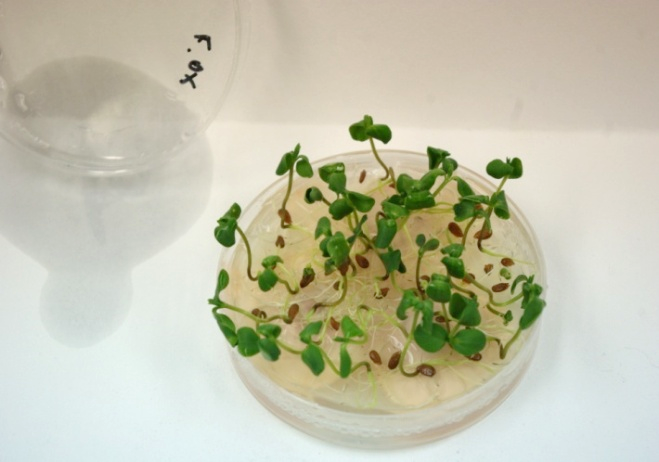 | 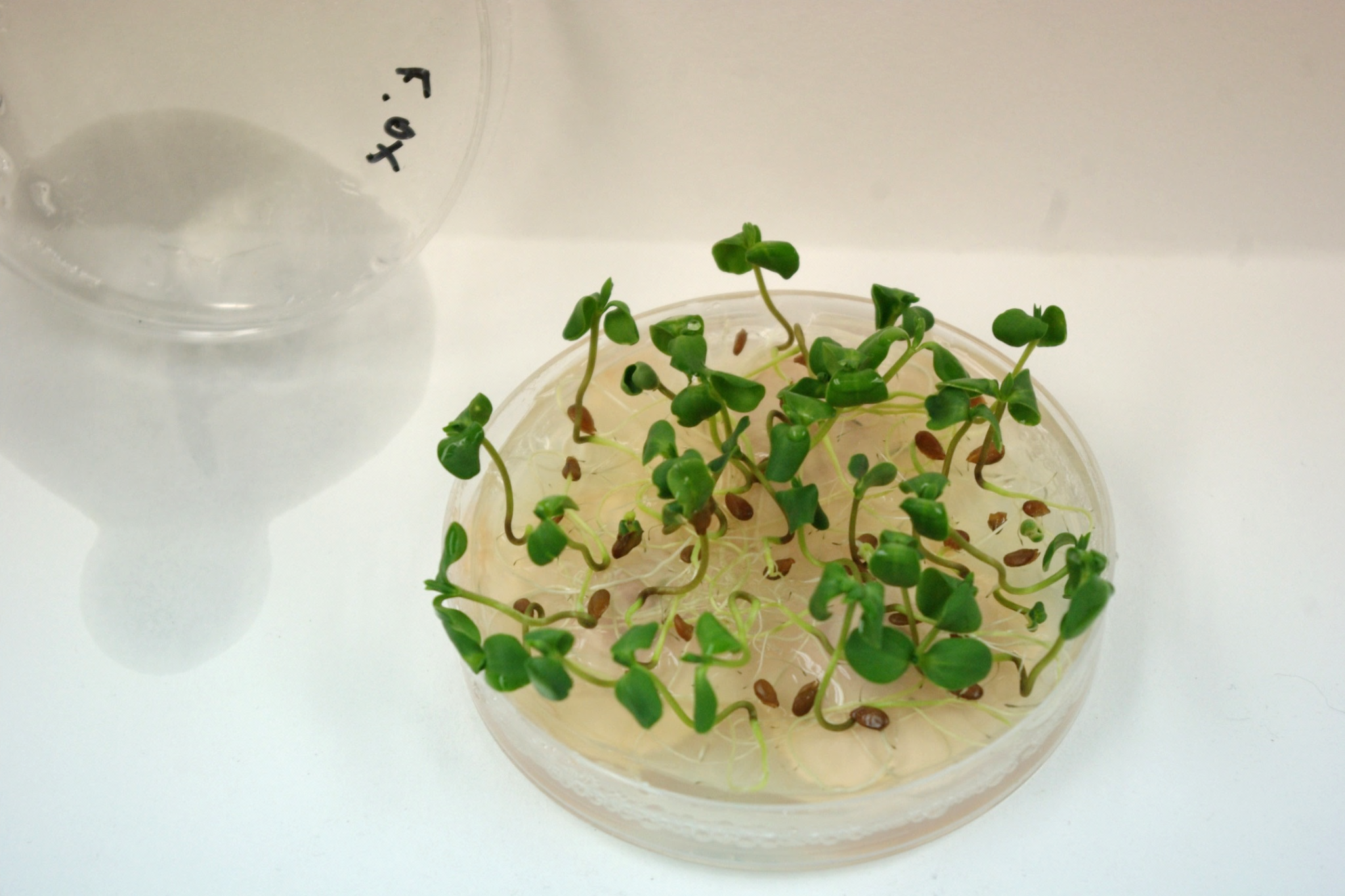 |
| 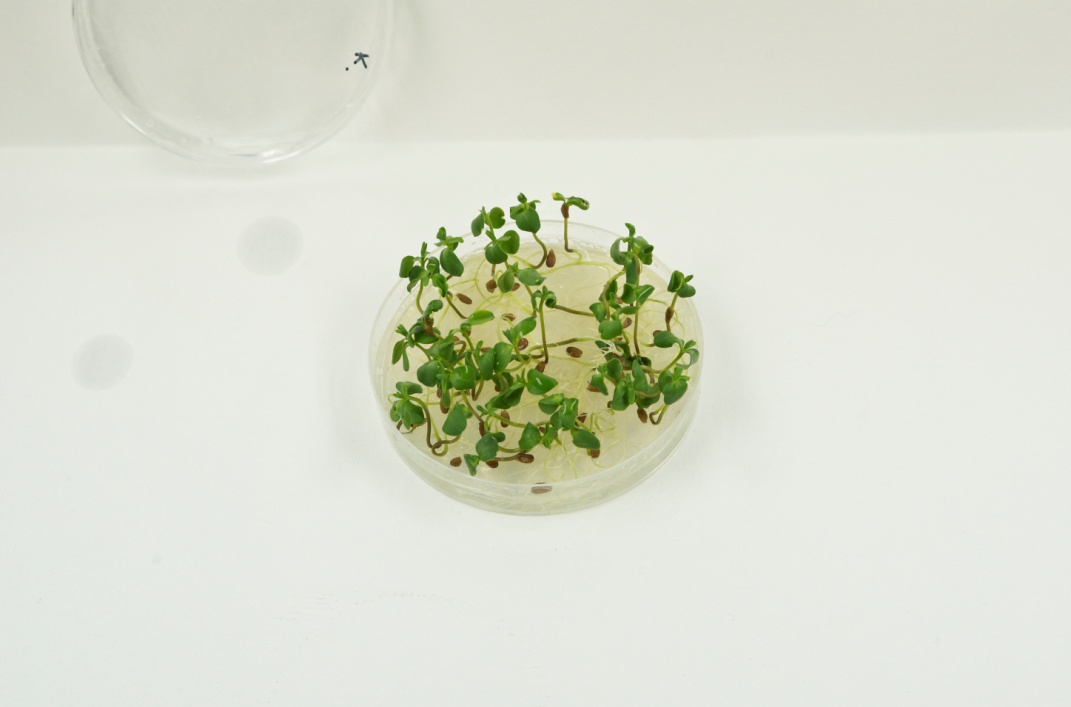  **48 HOURS OF THE INCUBATION** | 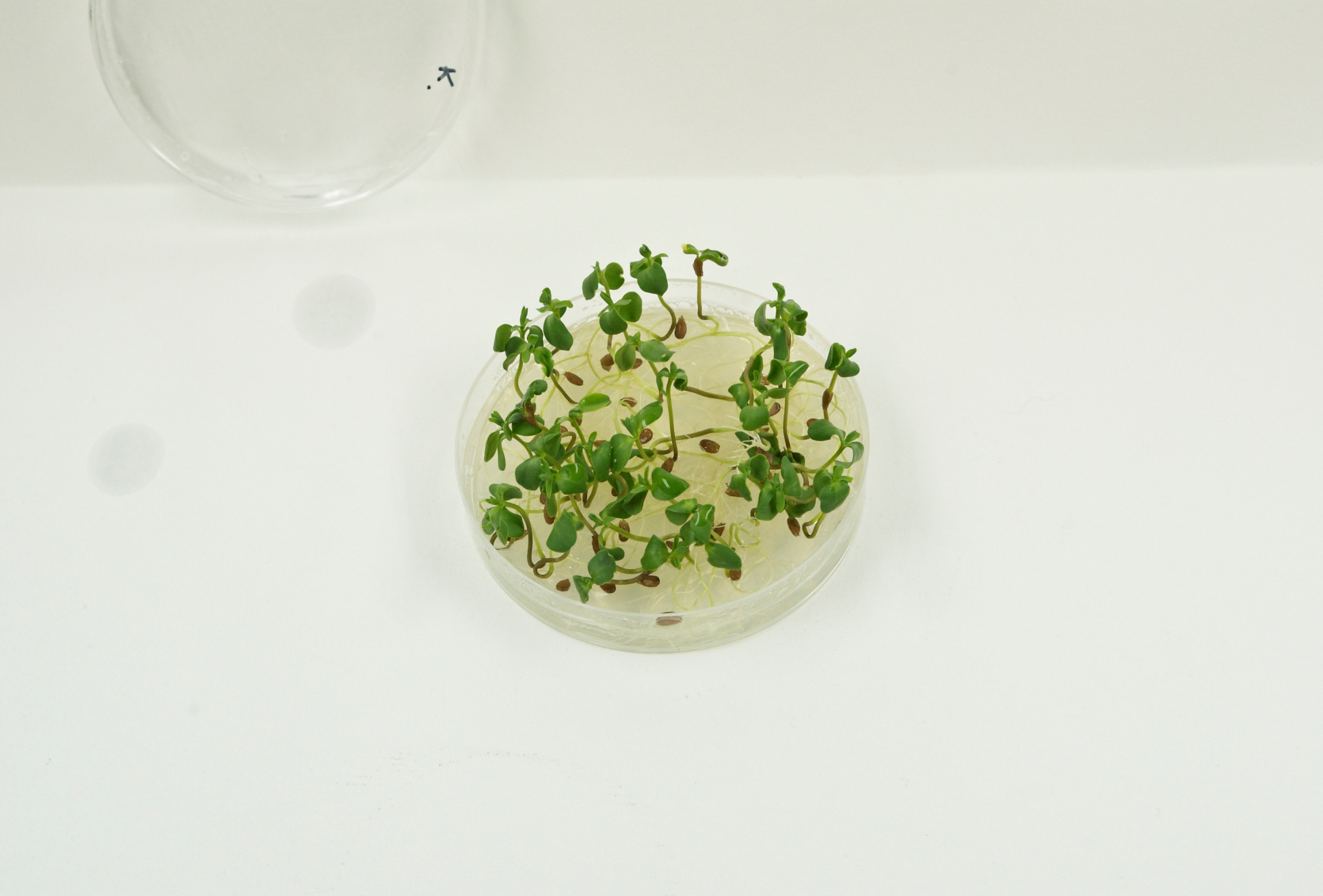 | 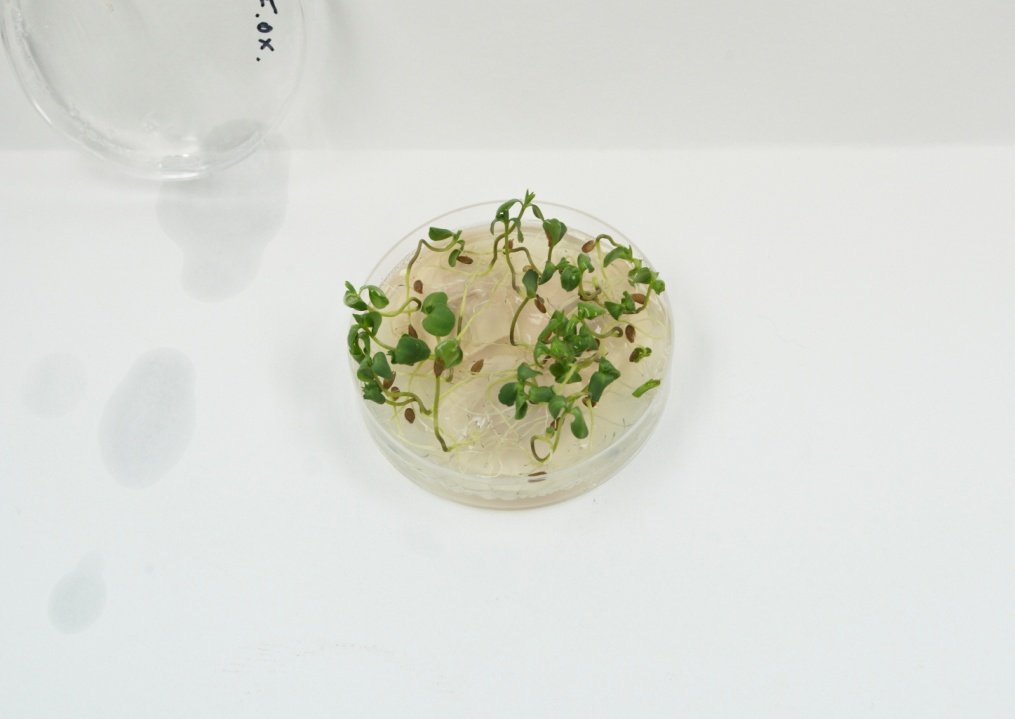 | 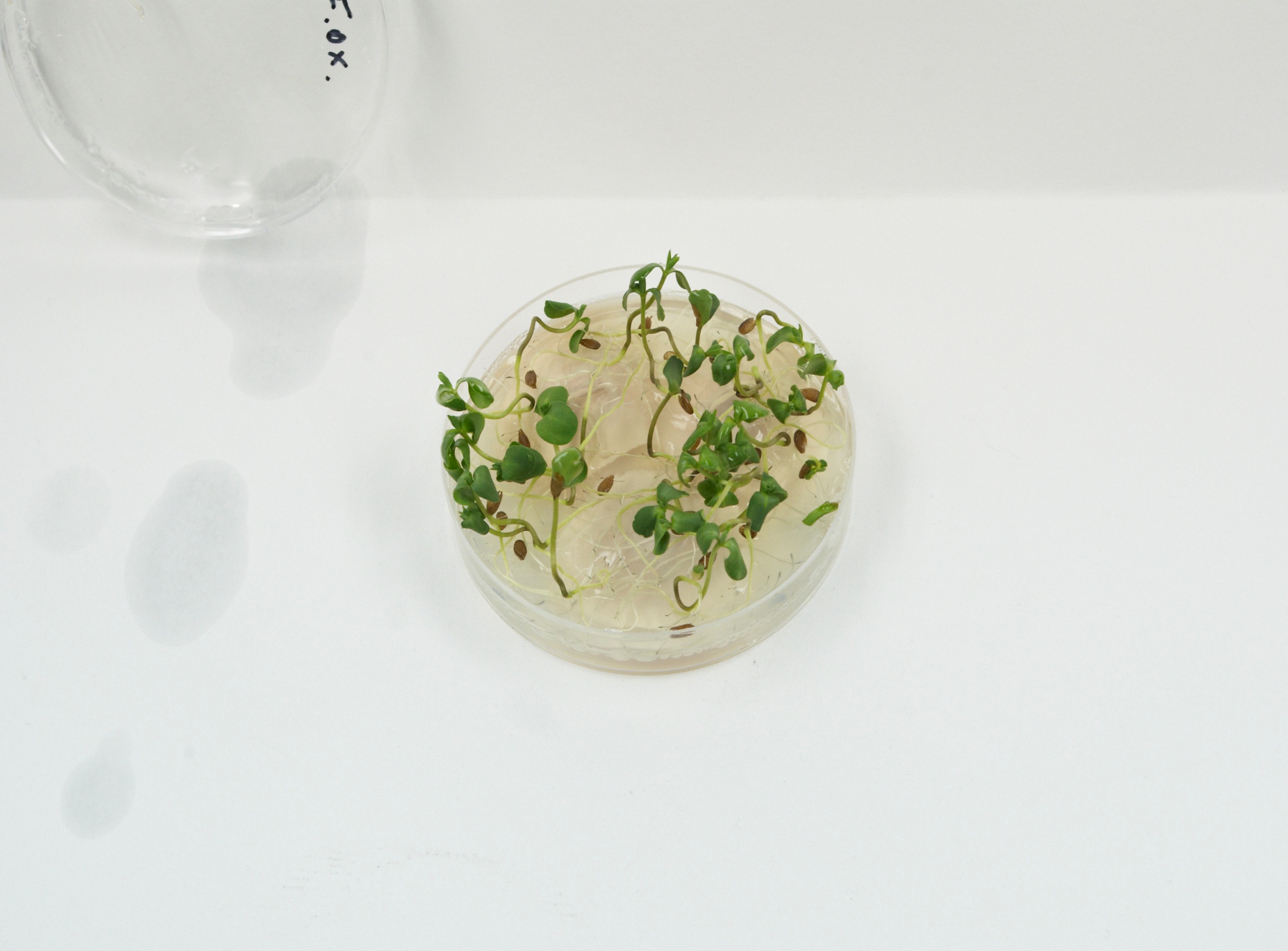 |
