# Supplementary figures and images for: Evaluation of the significance of cell wall polymers in flax infected with a pathogenic strain of Fusarium oxysporum
Source: BMC Plant Biol. 2016 Mar 22;16:75. doi: 10.1186/s12870-016-0762-z (PMC4804541; doi:10.1186/s12870-016-0762-z)

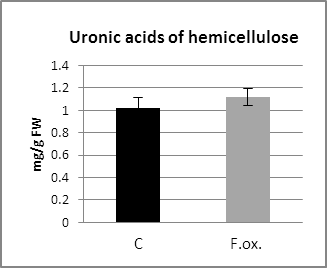

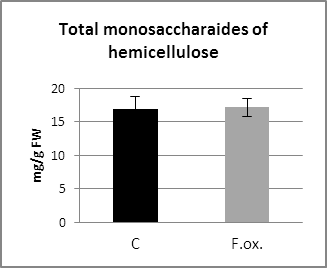

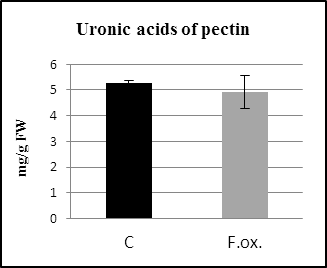

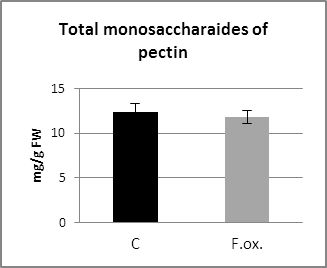


A

B

D

C

B

Additional figure 2

Supplement: Additional file 2: Figure S2. — The content of total uronic acids and total monosaccharides in hemicellulose and pectin fraction in flax seedlings infected with Fusarium oxysporum. Changes in total uronic acids (Additional file 2: Figure S2A and C) and total monosaccharaides (Additional file 2: Figure S2B and D) amount of hemicellulose and pectin fraction estimated based on the amount of uronic acids (Fig. 3 b and d) and monosaccharides (Fig. 3c and e). K1SF – 1 M KOH soluble fraction; K4SF – 4 M KOH soluble fraction; WSF – water soluble fraction; CSF –CDTA soluble fraction; NSF –Na2CO3 soluble fraction. Data represent the mean ± SD from four independent measurements. The significance of the differences between the means was determined using Student’s t test (*- P < 0.05, **- P < 0.01). (DOC 955 kb) [file 12870_2016_762_MOESM2_ESM.doc]
