# Supplementary material for: Evaluation of the significance of cell wall polymers in flax infected with a pathogenic strain of Fusarium oxysporum
Source: BMC Plant Biol. 2016 Mar 22;16:75. doi: 10.1186/s12870-016-0762-z (PMC4804541; doi:10.1186/s12870-016-0762-z)
Supplement: Additional file 3: Table S1. — Primer sequences for real-time RT-PCR reactions. Primer sequences designed for real-time PCR: (A) Cellulose metabolism genes. (B) Hemicellulose metabolism genes (C) Pectin metabolism genes. (D) Lignin metabolism genes. (E) PR genes and actin. (DOCX 20 kb) [file 12870_2016_762_MOESM3_ESM.docx]

Additional table 1

1. CELLULOSE METABOLISM

| **GENE** | **FORWARD PRIMER** | **REVERSE PRIMER** |
| --- | --- | --- |
| CSL1 | GGTCGAAATCTTCTTCTCACG | CGAGAATAAAGAGAGTGCGG |
| CSL2 | CTTACTCGCCAGTCCAAG | GTCACGTTGAGAGTTGACAC |
| CSL3 | AGTAGTAGGCTCAAGTTCCGA | GAATGCTATGTTTAGAGACTGGAC |
| CSL4 | TAATGTTGGCATCTACCCTTTC | CATTTGATCTCTAGGACGGC |
| CSL5 | TCAATGTCGGCATCTACCC | TGGCTAGACCAATGAGGC |
| CEL1 | CCATTACCCAAAGCACGTC | CTCGGTGTAGTTGTAGTTCATTC |
| CEL2 | AAGAGGCAAGTGGACTAC | GCCGATTTATAATAAACCGAGC |

1. HEMICELLULOSE METABOLISM

| **GENE** | **FORWARD PRIMER** | **REVERSE PRIMER** |
| --- | --- | --- |
| GMT | GCTAGAGTGCCAAAGGTG | GACCGATGTCCGAGTTATG |
| GGT | GTGTTTCTGATTCGTAACTGC | GAGTAAGTAGATCAACGCCG |
| XXT | AGATCGATTATTGCAGGCT | GCATCACTGTCCATCCAC |
| XYN | ACAGCAAGGAAGTCTTAACTAC | TCACAGCTTTCATTAAGTCATCT |
| XYL b | AAAGAACACCAGGATCTAGC | CTGCATAGTTTCCTAGAAGAGTC |
| XYL a | CAAGTATGATGATCAGCCTTTC | ATCTCGTTCATGTCGATCC |
| GS | CAGTCTCCTGGCGTGTTA | CAACTTTGACTCCATCTATGC |
| MS | TGACTATCTCGATGGCACAC | GGCATTCCTACAGAGCCAA |
| GLS | GATAAGGCCTCCGGATAAC | ATGAATACAGTGAATCGGGAC |

1. PECTIN METABOLISM

| **GENE** | **FORWARD PRIMER** | **REVERSE PRIMER** |
| --- | --- | --- |
| GAE | CCGAGGTACTCTTTCCTGA | CATACTTCTCGTTTACGAGGAAT |
| GAU 1 | AATTTAAGTGGCTTAATTCATCCTAC | GTGATTGAGCATTGAAAGATACTTG |
| GAU 7 | TGCAATATCCAAGGCACAGTTA | AGACAATGATGGCTCTTAGG |
| ARAD | ATTAACCAAGTATCCCGGC | TACTCCGTCAGCATGCAG |
| RGXT | TCCTGCGCTTGATTCACA | GGATCTCCCAGCCAAAC |
| PMT | ACGATTCTTGATATAGGCTGT | AGAACCAATCATCGCCG |
| PME1 | GAGCTGGAACCACATGC | AGCCTTCACGTATATTACGC |
| PME3 | AGTGGGATGGCAACTTT | TGAACCGACCGGGAGTA |
| PME5 | CGTAGTGGGTGACAGATTTAT | GAGGGAGTGGACGTAGAG |
| PG | GGATCAAGACTGCTGTGG | CAACAGGGATCGCGTTAG |
| PLL | GAGCATGTGATCGTATGCAA | CCTGGAAATGGTAATGTCAGT |
| PL | AACTACGAGAGAATCAAAGGAAC | GGGAGTATTCAACTCTCCG |

1. LIGNIN METABOLISM

| **GENE** | **FORWARD PRIMER** | **REVERSE PRIMER** |
| --- | --- | --- |
| PAL | GTTCTG TTTGAA GCCAAT GT | TGTAAG CACTCC CGTCG |
| 4CL | GCAGAAATGAAGATCGTCG | GTATGTAACCACCCTTGCT |
| CHS | AAATGG GGAGAA TGGAAG GA | CGCACG ATTCAA ATAGTG AGA |
| HCT | GTCGATATTCAAGCTGACCC | GTGGCGATGTACAGTTTAGT |
| C3H | CCATTGGAGTTCAAACCAGA | AGCAAGTGTCCCAACATAG |
| CCOACMT | CGGACAAGGACAACTACAT | ACGAAGTCCCTGTAGTACCTAA |
| COMT | CTCTTGGCTTCTTACTCTGTT | TGAGGACTTTGTCCTGGTT |
| SAD | CTACCTACGGAGGCTACT | GCTTGTCTAGCCCATAGAAC |
| GT | ATGCAGTGTGCATTCCAT | CGAATCGGAACGAAGGTAG |

1. PR GENES and ACTIN

| **GENE** | **FORWARD PRIMER** | **REVERSE PRIMER** |
| --- | --- | --- |
| β-1,3-GLU1 | CTAGGCAGCGTGAAAGC | CGTCGAAGAGGTTGGTG |
| β-1,3-GLU2 | GATCTGGTCAAAGAGGTTGGTATAA | GTCCTTCTTCTTCCTCGATG |
| CHIT | CATCCAATGAATGGCCTT | GGCTGTTCGGAATGATATCTC |
| ACT | CCGGTGTTATGGTTGGAAT | TGTAGAAAGTGTGATGCCAAA |
